# Alpha-mangostin inhibits viral replication and suppresses nuclear factor kappa B (NF-κB)-mediated inflammation in dengue virus infection

**DOI:** 10.1038/s41598-022-20284-7

**Published:** 2022-09-27

**Authors:** Mayuri Tarasuk, Pucharee Songprakhon, Thaweesak Chieochansin, Kornkan Choomee, Kesara Na-Bangchang, Pa-thai Yenchitsomanus

**Affiliations:** 1 Graduate Program in Bioclinical Sciences, Chulabhorn International College of Medicine, Thammasat University, Khlong Luang, Pathum Thani Thailand; 2 Division of Molecular Medicine, Research Department, Faculty of Medicine Siriraj Hospital, Mahidol University, 2 Wanglang Road, Bangkoknoi, Bangkok Thailand; 3 Drug Discovery and Development Center, Thammasat University, Khlong Luang, Pathum Thani Thailand

**Keywords:** Infectious diseases, Viral infection, Virology

## Abstract

Severe dengue virus (DENV) infection results from viral replication and dysregulated host immune response, which trigger massive cytokine production/cytokine storm. The result is severe vascular leakage, hemorrhagic diathesis, and organ dysfunction. Subsequent to previously proposing that an ideal drug for treatment of DENV infection should efficiently inhibit both virus production and cytokine storm, we discovered that α-mangostin (α-MG) from the pericarp of the mangosteen fruit could inhibit both DENV infection and cytokine/chemokine production. In this study, we investigated the molecular mechanisms underlying the antiviral and anti-inflammatory effects of α-MG. Time-of-drug-addition and time-of-drug-elimination studies suggested that α-MG inhibits the replication step of the DENV life cycle. α-MG inhibited polymerization activity of RNA-dependent RNA polymerase (RdRp) with IC50 values of 16.50 μM and significantly reduced viral RNA and protein syntheses, and virion production. Antiviral and cytokine/chemokine gene expression profiles of α-MG-treated DENV-2-infected cells were investigated by polymerase chain reaction array. α-MG suppressed the expression of 37 antiviral and cytokine/chemokine genes that relate to the NF-κB signaling pathway. Immunofluorescence and immunoblot analyses revealed that α-MG inhibits NF-κB nuclear translocation in DENV-2-infected cells in association with reduced RANTES, IP-10, TNF-α, and IL-6 production. These results suggest α-MG as a potential treatment for DENV infection.

## Introduction

Dengue virus (DENV) infection is the most widespread mosquito-borne viral disease, and it is caused by any one of the four dengue virus serotypes (DENV-1-4). The global incidence of DENV infection has increased dramatically in recent decades, and approximately half of the world population is now at risk. The World Health Organization estimates that there are 100–400 million DENV infections per year^1^. Most of those infected with DENV are asymptomatic or have mild illness, and about 5% of DENV-infected patients develop severe life-threatening disease^2^. Severe DENV infection is potentially fatal due to plasma leakage, fluid accumulation, respiratory distress, hemorrhagic diathesis, severe bleeding, organ dysfunction, and organ failure. Although, a vaccine to prevent dengue infection (Dengvaxia^®^; Sanofi Pasteur, Lyon, France) has been licensed, it is not widely used owing to safety concerns^3^. To date, no specific antiviral agents have been developed and introduced to treat DENV infection. Effective drugs to treat DENV infection are, therefore, urgently needed to prevent disease progression to severe disease, and for controlling the spread of the virus.

DENV contains approximately 11 kb of positive single-stranded genomic RNA that encodes a single polypeptide. The polyprotein is cleaved by both viral and host proteases to generate three structural proteins (capsid [C], pre-membrane/membrane [prM/M], and envelope [E]), and seven non-structural (NS) proteins (NS1, NS2A, NS2B, NS3, NS4A, NS4B, and NS5). The structural proteins form the viral particles, and the non-structural proteins participate in the replication of the RNA genome, virion assembly, and regulation of the host immune response^4^. It has been reported that the immune-dominant envelope protein (E protein) domain III region (EDIII) of DENV can trigger inflammatory responses. The EDIII protein induces the release of proinflammatory cytokines, such as IL-1β and TNF-α, in THP-1 cells via the NF-κB pathway, which may associate with increased inflammation during severe DENV infection^5^. NS1 is another protein that is involved in early viral RNA replication and increased proinflammatory cytokine production. During DENV infection, NS1 is secreted in the blood stream where it interacts with various immune mediators and cell types, which leads to increased proinflammatory cytokine production^6^. NS2B-NS3, NS4B, and NS5 are incorporated into the replication complex and play important roles during viral replication^7^. The NS5 protein plays a key role in both DENV replication and in the modulation of host immune response. The NS5 protein is the most highly conserved protein across all four DENV serotypes with 67–82% sequence identity^8^. NS5 has the enzymatic activities responsible for the replication of the viral genome. NS5 contains N-terminal methyltransferase (MTase) and the C-terminal RNA-dependent RNA polymerase (RdRp) catalytic domain, which are required for capping and synthesis of the RNA genome, respectively^9^. Both of these enzymatic activities in DENV NS5 are currently being investigated as targets for developing novel anti-DENV drugs. Moreover, NS5 is involved in both inhibition of the antiviral response and stimulation of chemokine production. NS5 inhibits interferon-α signaling, which is critical for innate antiviral responses^10^. In contrast, NS5 increases the production of chemokines, including interleukin-8 (IL-8), RANTES, MIP-1α, and MIP-1β, which may contribute to viral replication, as well as to the inflammatory components of DENV^11^. The researchers also reported that DENV NS5-induced RANTES expression is mediated via the NF-κB signaling pathway. NS5 translocates into the nucleus and interacts with the death-domain-associate protein (Daxx), which normally interacts with NF-κB, to increase RANTES production^12,13^. DENV NS4B and NS5 also induce IL-6, IL-8, and TNF-α production in monocytes, which contributes to the pathogenesis of severe DENV infection^14^.

The pathogenic mechanisms of DENV infection are complicated since they involve host immune response and multiple viral factors. Viral load correlates with disease severity, and antibody-dependent enhancement (ADE) in secondary infection enhances viral entry into host cells leading to increased viral load and immune activation associated with disease severity^15,16^. Aberrant inflammatory responses have been identified in DENV-infected patients. A number of host inflammatory biomarkers, including GM-CSF, IFN-γ, IL-10, IL-15, IL-8, MCP-1, IL-6, MIP-1β, and TNF-α, were increased in comparison to healthy controls. Four cytokines (IFN-γ, GM-CSF, IL-10, and MIP-1β) were reported to be significantly correlated with disease severity^17^. Liver injury is commonly observed in severe DENV infection. The increase in the liver enzymes aspartate transaminase (AST) and alanine transaminase (ALT) appears to correlate with disease severity^18^. The pathogenesis of liver injury in DENV infection mainly involves direct viral cytopathic effects and immune‐mediated injury. Infected hepatocytes express IFN-α, IFN-β, RANTES, IL‐6, IL‐8, IL‐10, and IL‐12 via NF-κB. IFN-α and IFN-β enhance antiviral defense of nearby cells. RANTES, IL‐6, IL‐8, IL‐10, and IL‐12 attract NK cells, CD8, and CD4 lymphocytes that induce hepatocyte apoptosis^19^.

Since high viral titer and excessive cytokine production are both known to be associated with severe DENV infection, concurrent inhibition of both DENV replication and cytokine production should be an effective strategy for treating DENV infection. Here, we propose α-MG as a candidate agent for the treatment of DENV infection given its potent antiviral and anti-inflammatory activities. α-MG is the most abundant xanthone in mangosteen fruit (*Garcinia mangostana* Linn.), and it possesses broad biological activities, including anti-inflammatory, antiallergic, antiviral, antibacterial, antifungal, antiparasitic, antioxidant, and anticancer properties^20^. α-MG exerts potent antiviral effects against a variety of human pathogenic viruses, including human immunodeficiency virus (HIV), rotavirus, hepatitis C virus (HCV), DENV, and chikungunya virus (CHIKV)^21–25^. HIV protease and reverse transcriptase were reported to be potential targets of α-MG^21,26^. A recent study reported that α-MG inhibited CHIKV infection and replication via possible interaction with multiple CHIKV target proteins, the envelope protein complex, and the nsP3 macrodomain^25^. Interestingly, α-MG was observed to suppress HCV replication by inhibition of NS5B RNA-dependent RNA polymerase (RdRp) activity^23^.

Moreover, the anti-inflammatory property of α-MG has been widely investigated in various inflammatory disease models. α-MG protects rat articular chondrocytes against IL-1β-induced inflammation, and reduces the progression of osteoarthritis in rat model by inhibiting the overexpression of inflammatory mediators via blocked NF-κB activation^27^. Similarly, α-MG was shown to attenuate brain inflammation induced by peripheral lipopolysaccharide administration in C57BL/6J mice by reducing the levels of the proinflammatory cytokine IL-6, cyclooxygenase-2 (COX-2), and the 18 kDa translocator protein (TSPO)^28^. In addition, α-MG showed hepatoprotective effect in lipopolysaccharide/D-galactosamine (LPS/D-GalN)-induced acute liver failure in mice. α-MG was reported to attenuate LPS/D-GalN-induced pathological liver injury by inhibiting toll-like receptor 4 (TLR4) expression and NF-κB activation to induce anti-inflammatory effect, and by activating nuclear factor-erythroid factor 2-related factor 2 (Nrf2) to induce antioxidant defense^29^.

In a previous study, our group demonstrated that α-MG exerts both antiviral and anti-inflammatory effects against DENV infection. α-MG could efficiently reduce DENV-infected cells and viral production in all four DENV serotypes. We also previously found and reported that treatment of DENV-infected cells with α-MG also significantly reduced cytokine/chemokine expression in human hepatocellular carcinoma (HepG2) cells^24^. However, the mechanism of α-MG action has not yet been elucidated. Accordingly, we set forth to investigate the molecular mechanism underlying the antiviral and anti-inflammatory activities of α-MG against DENV infection. In the present study, we investigated the effect of α-MG on the DENV life cycle; we performed transcriptional profiling of host antiviral and anti-inflammatory gene expression in α-MG-treated DENV-2-infected HepG2 cells; we investigated the inhibitory effect of α-MG on NS5 RdRp activity; and, we evaluated the effects of α-MG on NS5 nuclear accumulation and NF-κB nuclear translocation, which associated with DENV-induced cytokine/chemokine gene expression.

## Results

### α-MG reduced DENV infection and virus production in DENV-2-infected HepG2 cells

**Figure 1:**
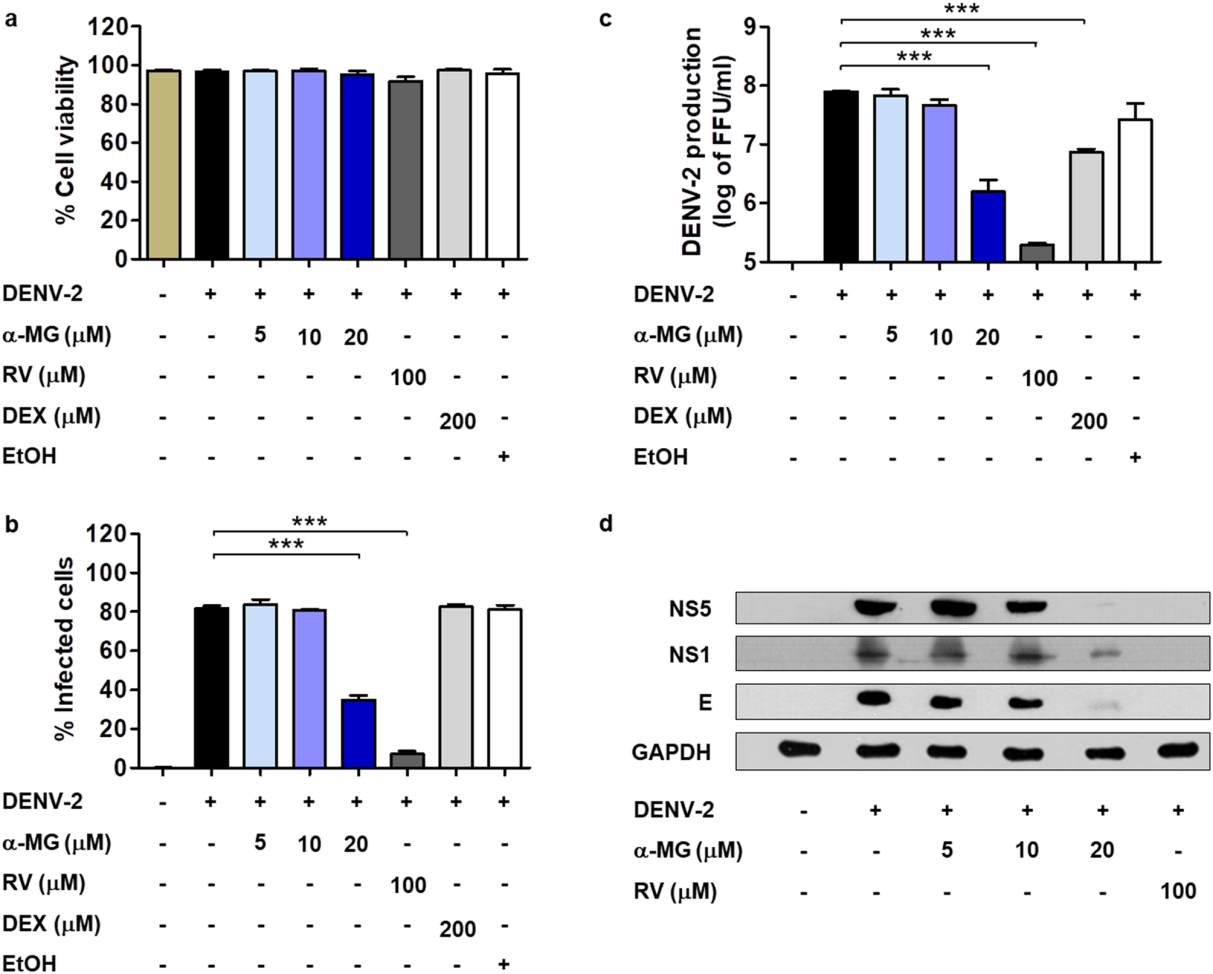
α-MG reduces DENV-2 infection, DENV-2 production, and downregulation of DENV-2 protein expression. HepG2 cells were infected with DENV-2 at a MOI of 5 and treated with α-MG, ribavirin (RV), dexamethasone (DEX), or ethanol (EtOH) solvent at the indicated concentrations for 24 h. (**a**) The percentages of cell viability were evaluated by trypan blue exclusion assay. (**b**) The percentages of infected cells were determined by flow cytometry using the mouse monoclonal anti-DENV-E antibody (4G2). (**c**) DENV-2 production in culture supernatants was determined by FFU assay. The results were collected from three independent experiments, and the mean ± SD values were calculated and plotted. Statistical differences compared to the control group were calculated by one-way ANOVA and Tukey’s HSD test (*p < 0.05, **p < 0.01, ***p < 0.001). (**d**) The expression levels of NS5, NS1, and E protein after treatment with α-MG as examined by immunoblot analysis using the anti-NS5, anti-NS1, and anti-E antibodies, respectively.

To confirm the antiviral activity of α-MG, HepG2 cells were infected with DENV-2 at an MOI of 5 and subsequently treated with α-MG at 5, 10, or 20 μM or antiviral control—ribavirin (RV) at 100 μM or anti-inflammatory control—dexamethasone (DEX) at 200 μM. Cell viability, percentage of infected cells, and DENV-2 production were determined at 24 h post-treatment by trypan blue exclusion assay, flow cytometry, and FFU assay, respectively (Fig. 1a–c). The results showed that α-MG had a potent inhibitory effect on DENV infection and production in a dose-dependent manner (Fig. 1b,c). α-MG at 20 μM significantly reduced the proportion of DENV-2-infected cells to 35% (*p* < 0.001), while the proportion of infected cells in the untreated control was 82% (Fig. 1b). At the same time, DENV-2 production in HepG2 cells exposed to 20 μM of α-MG was significantly reduced by more than 100-fold reduction (*p* < 0.001) when compared to that of untreated DENV-2-infected cells (Fig. 1c). DENV-2 NS5, NS1, and E proteins were detected by immunoblot analysis after treatment with α-MG. As expected, all DENV-2 proteins were downregulated after treatment with α-MG (Fig. 1d and Fig. S1).

### α-MG inhibited the viral replication step in the DENV life cycle

**Figure 2:**
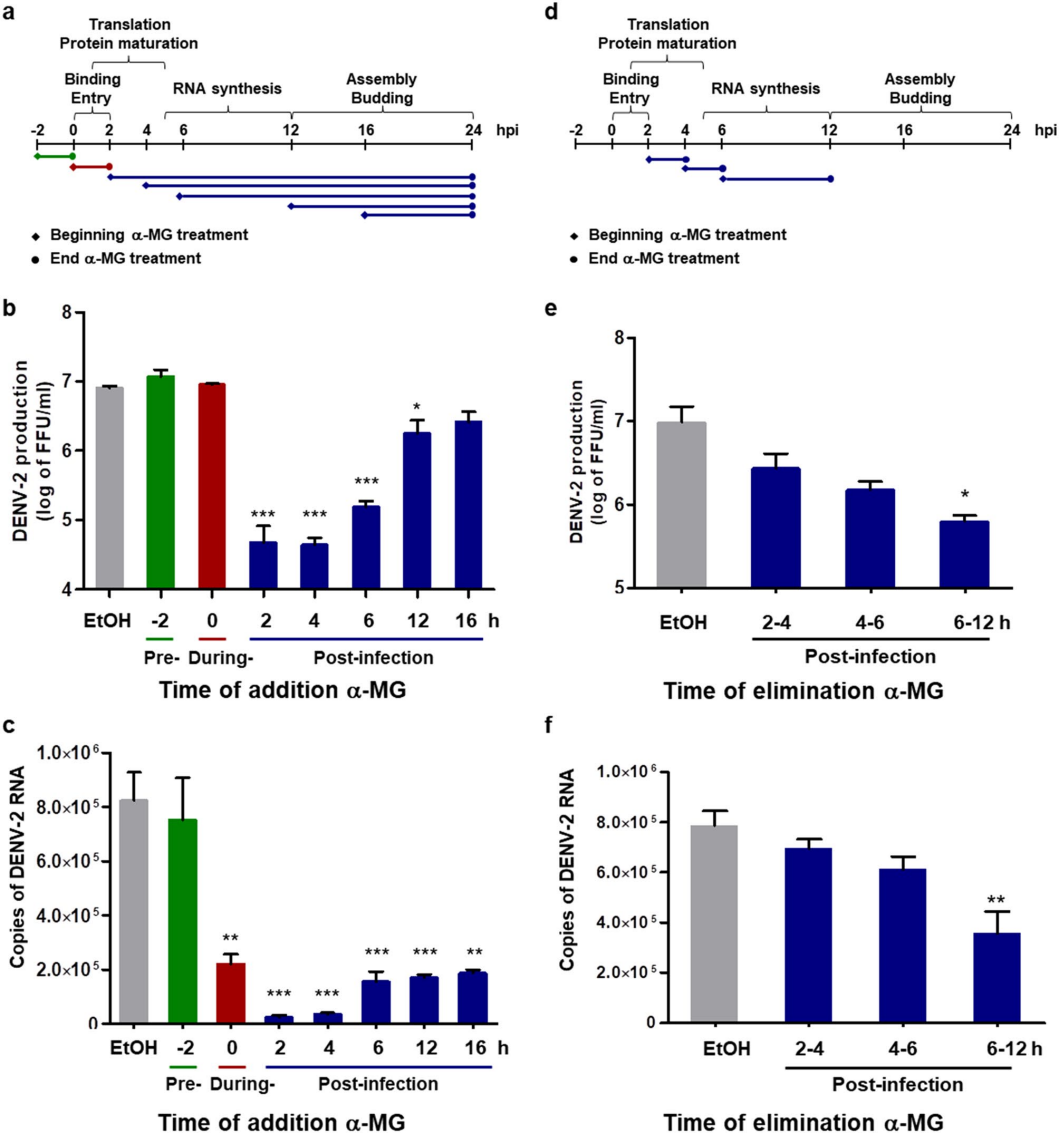
α-MG inhibits the replication steps in the DENV life cycle. (**a**) Horizontal flow chart of the experiment set-up for time-of-drug-addition assay. Twenty μM of α-MG was added at 2 h before infection, at 0–2 h during infection, and at 2, 4, 6, 12, and 16 h post-infection (hpi). (**b**) At 24 hpi, the concentration of released viruses in the supernatant was determined by FFU assay. (**c**) DENV-2 NS1 gene replication was quantitated by qRT-PCR. (**d**) Horizontal flow chart of the experiment set-up for time-of-drug-elimination assay. Twenty μM of α-MG was added during 2–4, 4–6, and 6–12 hpi. (**e**) At 24 hpi, the concentration of released viruses in the supernatant was determined by FFU assay. (**f**) DENV-2 NS1 gene replication was quantitated by qRT-PCR. The results show the mean ± SD values from three independent experiments. Differences were assessed by one-way ANOVA and Tukey’s HSD test (*p < 0.05, **p < 0.01, ***p < 0.001).

To determine the step of the DENV life cycle that was inhibited by α-MG, time-of-drug-addition and time-of-drug-elimination assays were conducted. During a single DENV life cycle, virus particles attach to receptors on the host cells and enter into the cell within 2 h. Viral proteins are translated from genomic RNA during the first 1–5 h post-infection (hpi). Viral RNA synthesis occurs after 5 hpi, and progeny virions are assembled and released after 12 hpi^30^. For time-of-drug-addition assay, 20 μM of α-MG was added at 2 h preinfection, at 0–2 h during infection, and at 2, 4, 6, 12, and 16 hpi (Fig. 2a). At 24 hpi, released viruses in the supernatant were quantitated by FFU assay. The addition of α-MG at 2, 4, 6, and 12 hpi significantly reduced the virus production, while the presence of α-MG at 2 h preinfection, 0–2 h during infection, and 16 hpi did not show any effect on virus production (Fig. 2b). The intracellular viral RNA copy number at different α-MG addition time points was quantitated by qRT-PCR. The addition of α-MG at 0–2 h during infection, and at 2, 4, 6, 12, and 16 hpi significantly reduced the viral copy number, whereas α-MG treatment at 2 h preinfection did not have any significant effect on the viral copy number (Fig. 2c).

The target of α-MG at the DENV replication step was examined by time-of-drug-elimination assay. α-MG at 20 μM was added during 2–4, 4–6, and 6–12 hpi (Fig. 2d). At 24 hpi, released viruses in the supernatant were determined by FFU assay, and the intracellular viral RNA copy number was quantitated by qRT-PCR. The results showed that α-MG treatment during 6–12 hpi significantly reduced virus production in the supernatant, and significantly reduced the viral copy number in DENV-2-infected HepG2 cells (Fig. 2e,f). Although the level of virus production and viral copy number were reduced when DENV-2-infected HepG2 cells were exposed to α-MG during 2–4 or 4–6 hpi, those values were not significantly different from those of the untreated control. Thus, α-MG showed significant effect at the stage of viral RNA synthesis, but did not exert significant effect at the stages of viral attachment and entry, or viral protein translation.

### α-MG inhibited RdRp activity

**Figure 3:**
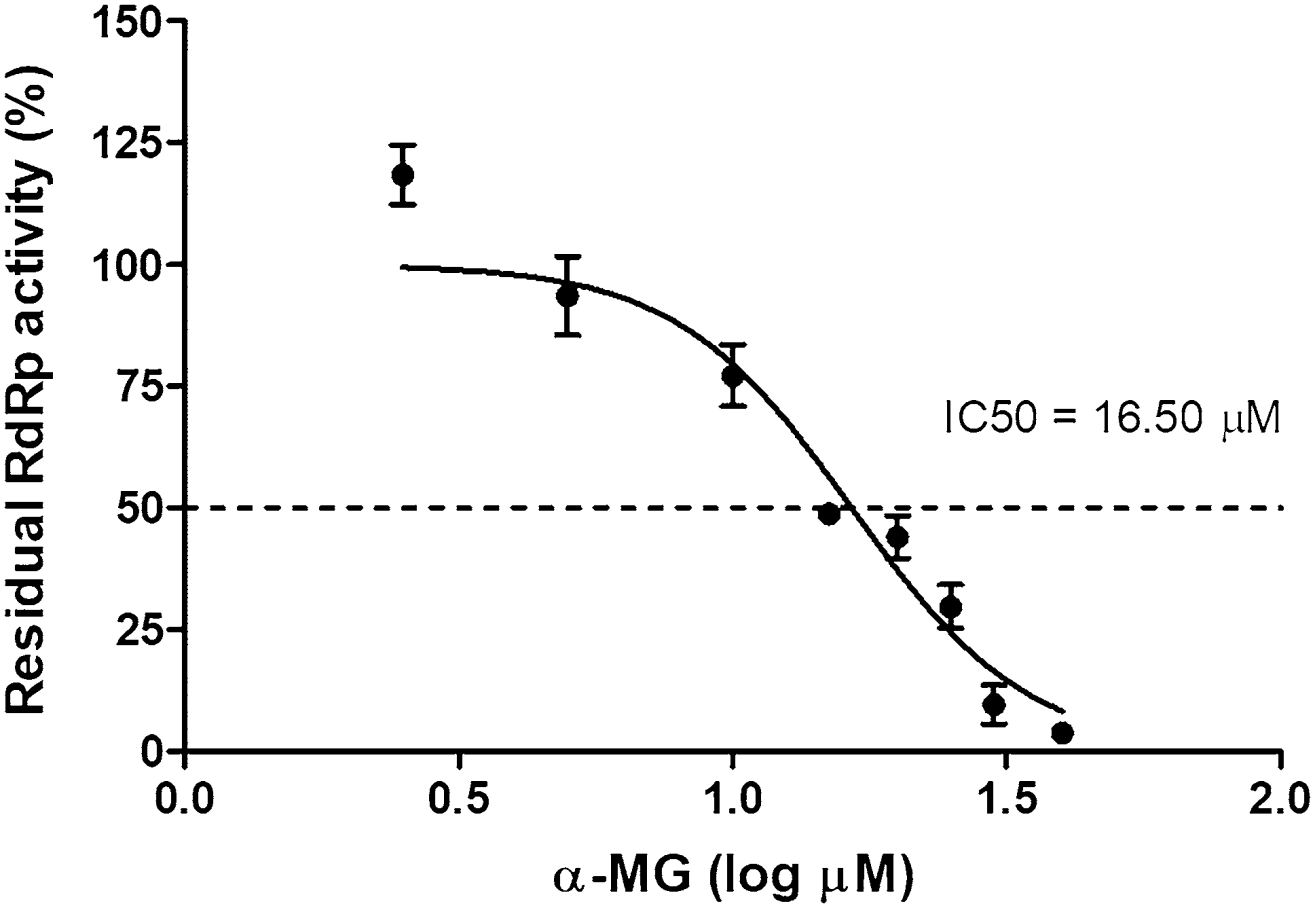
α-MG inhibits the activity of DENV-2 RdRp. The inhibitory effect of α-MG on the activity of DENV-2 RdRp was performed by in vitro fluorescence-based transcription assay. Dose–response curve shows inhibition by α-MG (0–40 μM) against the activity of DENV-2 RdRp at 2 μM. The positive and negative controls were treated with EtOH in the presence and absence of poly(C) templates, respectively. IC50 values were obtained by non-linear regression analysis using GraphPad Prism. The results show the mean ± SD values from three independent experiments.

To investigate the molecular mechanisms underlying the antiviral effect of α-MG, the RdRp domain of DENV-2 NS5 was expressed in mammalian cells using lentiviral expression system and purified by TALON™ Metal Affinity Resin under native conditions. The purified recombinant RdRp was verified by immunoblot analysis using anti-HA antibody to detect HA-tagged RdRp. The predicted molecular weight of this protein is 76 kDa (Fig. S2). Polymerase activity of DENV-2 RdRp was measured by in vitro fluorescence-based transcription assay, where the formation of dsRNA from poly(C) templates was detected with PicoGreen dye^31^. To determine the inhibitory effect of α-MG on the activity of DENV-2 RdRp, RdRp assay was performed in the presence of different concentrations of α-MG. Dose-dependent inhibitory response curve (0–40 μM) was generated to establish the IC50 value. The results showed that α-MG markedly inhibited DENV-2 RdRp activity with IC50 of 16.50 μM (Fig. 3).

### α-MG reduced cytokine/chemokine transcription and secretion induced by DENV-2 infection

**Figure 4:**
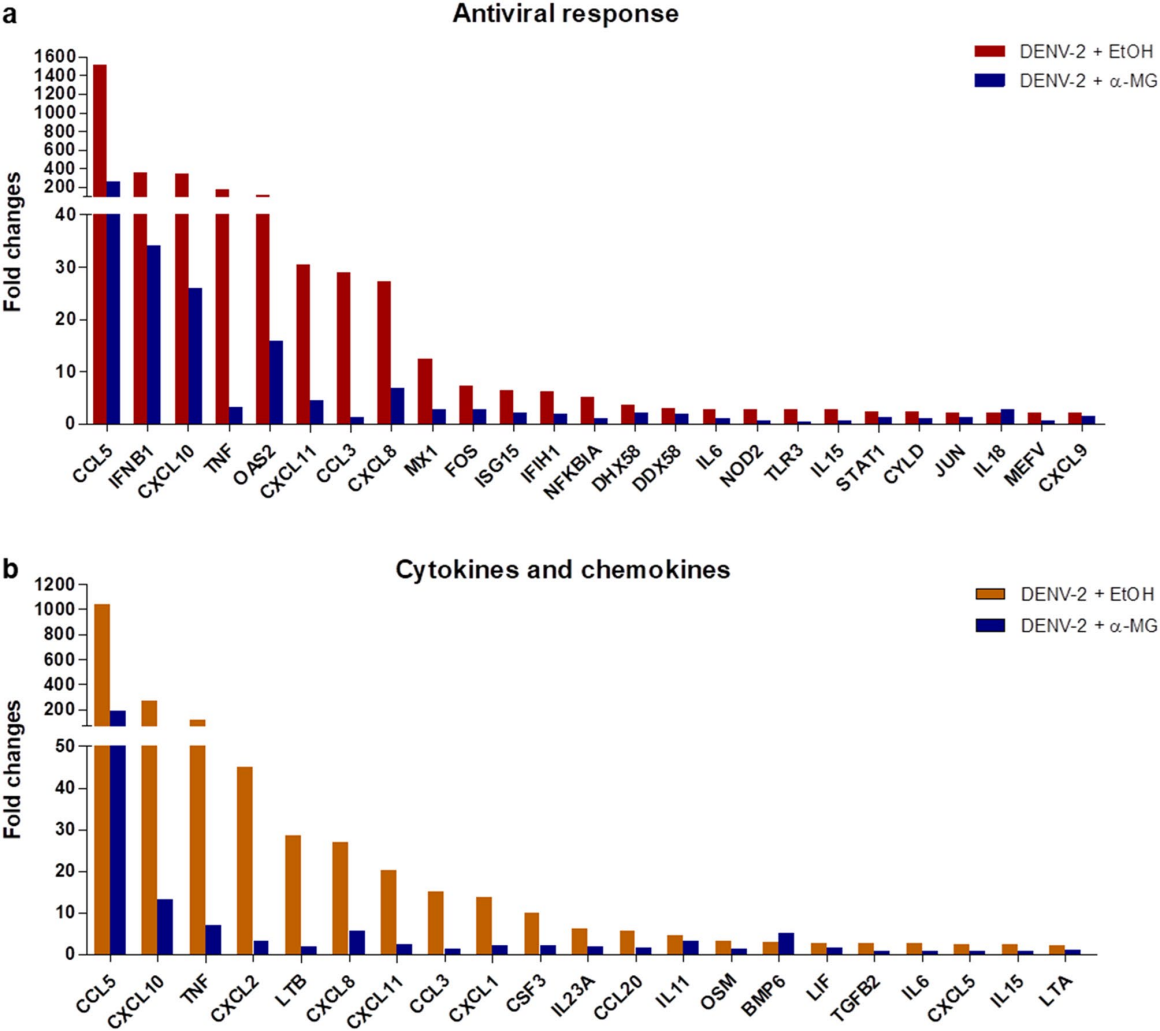
Real-time reverse transcription polymerase chain reaction (real-time RT-PCR) array profile of (**a**) antiviral genes, and (**b**) cytokine and chemokine genes in DENV-infected HepG2 cells in the presence or absence of 20 μM α-MG.

**Figure 5:**
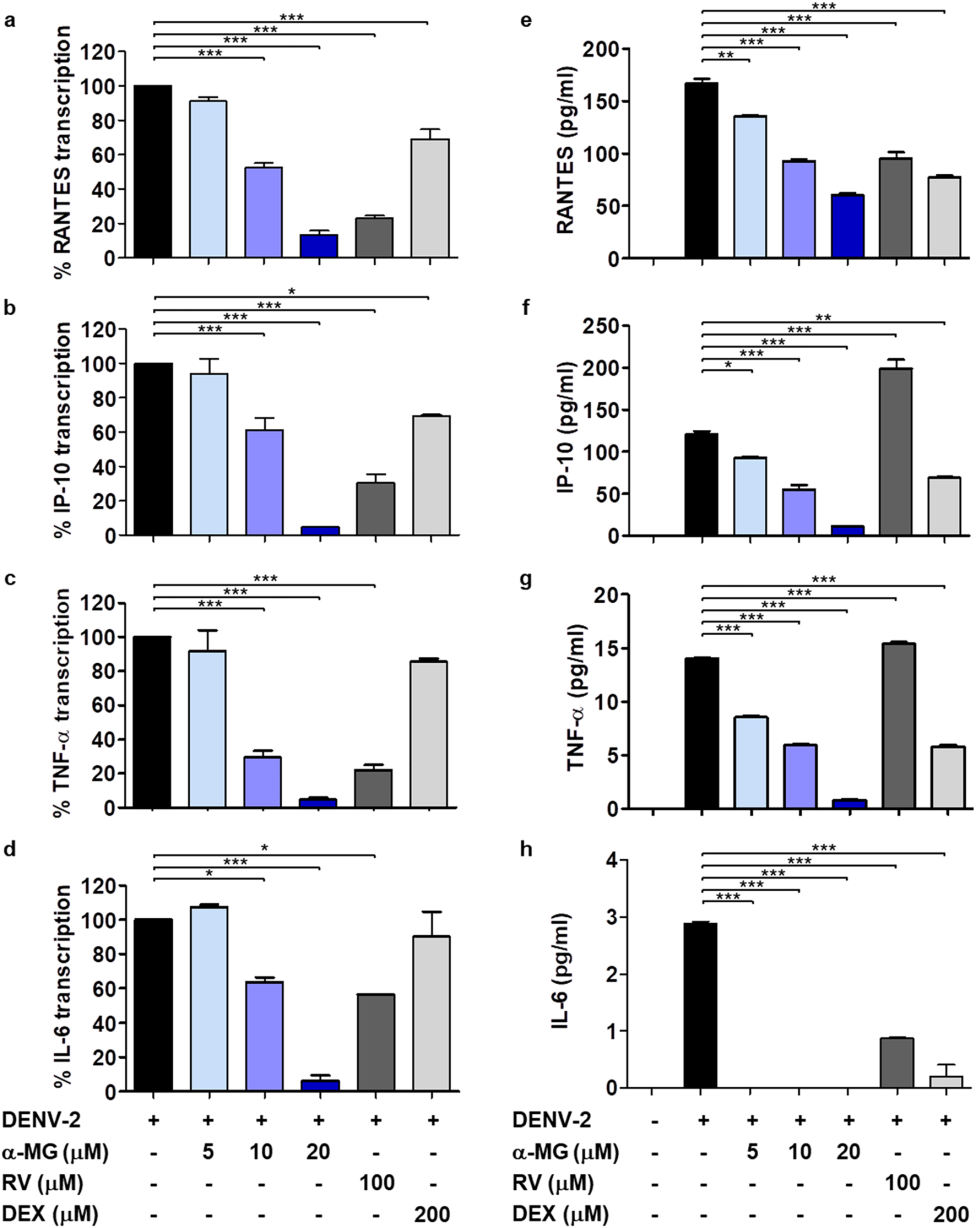
α-MG reduces cytokine/chemokine production in DENV-infected HepG2 cells. HepG2 cells were infected with DENV-2 at a MOI of 5 and treated with either α-MG, RV, DEX, or EtOH solvent at the indicated concentrations for 24 h. (**a**–**d**) The concentrations of transcription of RANTES, IP-10, TNF-α, and IL-6 in DENV-2-infected HepG2 cells were determined by qRT-PCR using different sets of specific primers. The transcription level of each cytokine/chemokine in untreated DENV-infected cells was defined as 100%. (**e**–**h**) The culture supernatants were collected and the cytokine/chemokine levels of RANTES, IP-10, TNF-α, and IL-6 were determined by flow cytometry using multiplexed bead-based immunoassays. The results were obtained from three independent experiments, and the results are expressed in mean ± standard deviation. One-way ANOVA followed by Tukey’s HSD test were used to evaluate for differences in cytokine/chemokine production levels as compared to control untreated DENV-infected HepG2 cells (*p < 0.05, **p < 0.01, ***p < 0.001).

To determine the potential anti-DENV targets and the mechanisms of α-MG to inhibit DENV infection and cytokine/chemokine production, the expression of antiviral- and anti-inflammatory-related genes in response to α-MG were investigated by real-time RT-PCR array. HepG2 cells were infected with DENV-2 and treated with/without 20 μM of α-MG for 24 h. Total RNA was extracted and mRNAs were reverse-transcribed into cDNA before amplification by real-time PCR array. Non-DENV-infected HepG2 cells were used as mock control. Supplementary Tables 1 and 2 describe the genes in the antiviral and cytokine/chemokine pathways that exhibited changes in expression greater than twofold compared to those of the untreated DENV-2 infected cells. The expression of almost all elevated genes was suppressed by α-MG, as shown in Supplementary Tables 1 and 2, and Fig. 4. CCL5 (RANTES), CXCL10 (IP-10), TNF-α, and IL-6 were selected as representatives of DENV-induced cytokines/chemokines to further investigate the effects of α-MG in DENV infection by qRT-PCR. Similarly, the expressions of RANTES, IP-10, TNF-α, and IL-6 were upregulated in DENV-2-infected cells when compared to those of the mock infected cells. The data are expressed as the percentage of transcription relative to that of the untreated DENV-2 infected cells, which was defined as 100%. All tested cytokine/chemokine transcriptions were significantly suppressed by α-MG (*p* < 0.001) (Fig. 5a–d). Treatment of DENV-2-infected cells with 20 μM of α-MG could significantly reduce the transcription of RANTES, IP-10, TNF-α, and IL-6 to 13%, 5%, 5%, and 6%, respectively.

To confirm the anti-inflammatory activity of α-MG, the secreted RANTES, IP-10, TNF-α, and IL-6 proteins in the culture supernatants after treatment of DENV-2-infected cells with α-MG were measured using multiplexed bead-based immunoassays. Similar to the cytokine/chemokine transcription results, α-MG significantly reduced RANTES, IP-10, and TNF-α protein secretion in a dose-dependent manner when compared to those of the untreated DENV-2-infected cells (Fig. 5e–g); however, IL-6 became undetectable after DENV-2-infected cells were treated with 5–20 μM of α-MG (Fig. 5h). Treatment of DENV-2-infected cells with DEX significantly reduced RANTES, IP-10, TNF-α, and IL-6 levels (Fig. 5e–h). Treatment of DENV-2-infected cells with RV significantly reduced RANTES and IL-6 levels (Fig. 5e,h), but—unexpectedly, the levels of IP-10 and TNF-α were not reduced (Fig. 5f,g).

### α-MG inhibited NF-κB-mediated cytokine/chemokine production

**Figure 6:**
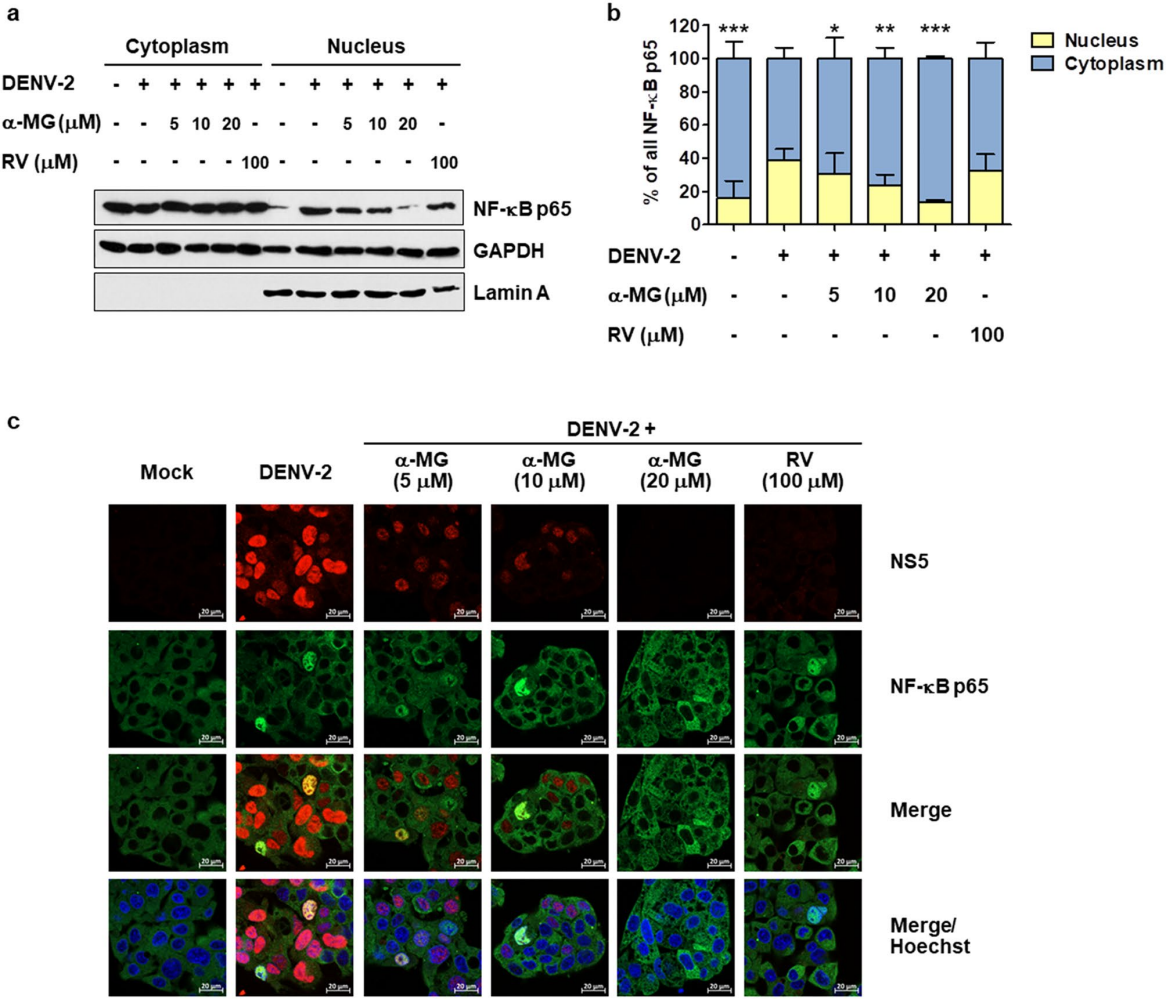
α-MG inhibits NF-κB nuclear translocation. HepG2 cells were infected with DENV-2 at a MOI of 5 and treated with 5, 10, or 20 μM of α-MG or 100 μM of RV. HepG2 cells that were not infected with DENV-2 were used as mock control. The cells were harvested at 24 h after treatment. Cytoplasmic and nuclear proteins were fractionated. (**a**) The expression of NF-κB p65 was determined by Western blotting using an anti-NF-κB p65 antibody. NF-κB p65 bands were scanned and quantified using ImageJ software. Lamin A was used as a protein marker of nuclear fraction. (**b**) The levels of NF-κB p65 were quantified, and the average values from three independent experiments were calculated after being normalized to the level of the GAPDH protein. The intracellular distribution of NF-κB p65 between the nucleus and cytoplasm was calculated from the levels of NF-κB p65 in the nucleus or cytoplasm divided by the total nuclear plus cytoplasmic levels of NF-κB p65. Two-way ANOVA and Bonferroni posttest were used to determine differences of NF-κB p65 levels between nuclear and cytoplasmic fractions and between treatment groups. (**c**) Immunofluorescence assay demonstrates the expression and intracellular localization of NF-κB p65 (green) and DENV NS5 (red). Hoechst stain was used for nuclear staining (blue). Images are representative of three independent experiments.

To characterize how α-MG inhibits DENV-2-induced cytokine/chemokine production, we first analyzed whether α-MG treatment could inhibit NF-κB activation in response of DENV infection using immunoblot analysis. HepG2 cells were infected with DENV-2 and then treated with either α-MG at 5, 10, or 20 μM or RV at 100 μM. At 24 h post-treatment, cytoplasmic and nuclear proteins were fractionated, followed by immunoblot analysis. Their intracellular distribution of NF-κB p65 was determined and quantified. As shown in Fig. 6a and Fig. S3, α-MG treatment markedly reduced the level of NF-κB p65 in nuclear fraction. Lamin A was used as the nuclear marker. Lamin A was detected only in the nuclear fraction, but not in the cytoplasmic fraction, which indicates that the subcellular fractionation of the nucleus versus the cytoplasm was complete. The levels of NF-κB p65 were calculated from three independent experiments after normalization to the levels of the GAPDH protein. Intracellular distributions of NF-κB p65 between the nucleus and cytoplasm were calculated from the levels of NF-κB p65 in the nucleus or cytoplasm divided by the total nuclear plus cytoplasmic levels of NF-κB p65. The percentages of nuclear and cytoplasmic NF-κB p65 are shown in Fig. 6b. The level of nuclear NF-κB p65 in the mock infected cells was 16.30%, but it was increased to 38.82% in the untreated DENV-2-infected cells. Nuclear NF-κB p65 levels were significantly decreased in the presence of 5, 10, and 20 μM of α-MG to 30.52%, 23.66%, and 13.54%, respectively, compared to those of the untreated DENV-2-infected cells. The effects of α-MG on DENV-induced nuclear translocation of NF-κB p65 were further analyzed using immunofluorescence assay. NS5 was predominantly located in the nucleus of untreated DENV-infected cells where it is thought to play a role in the suppression of host antiviral responses. Nuclear accumulation of NS5 in the DENV-2-infected cells was decreased in response to α-MG treatment (Fig. 6c). Consistent with the immunoblot result, α-MG markedly inhibited DENV-induced nuclear translocation of NF-κB p65.

**Figure 7:**
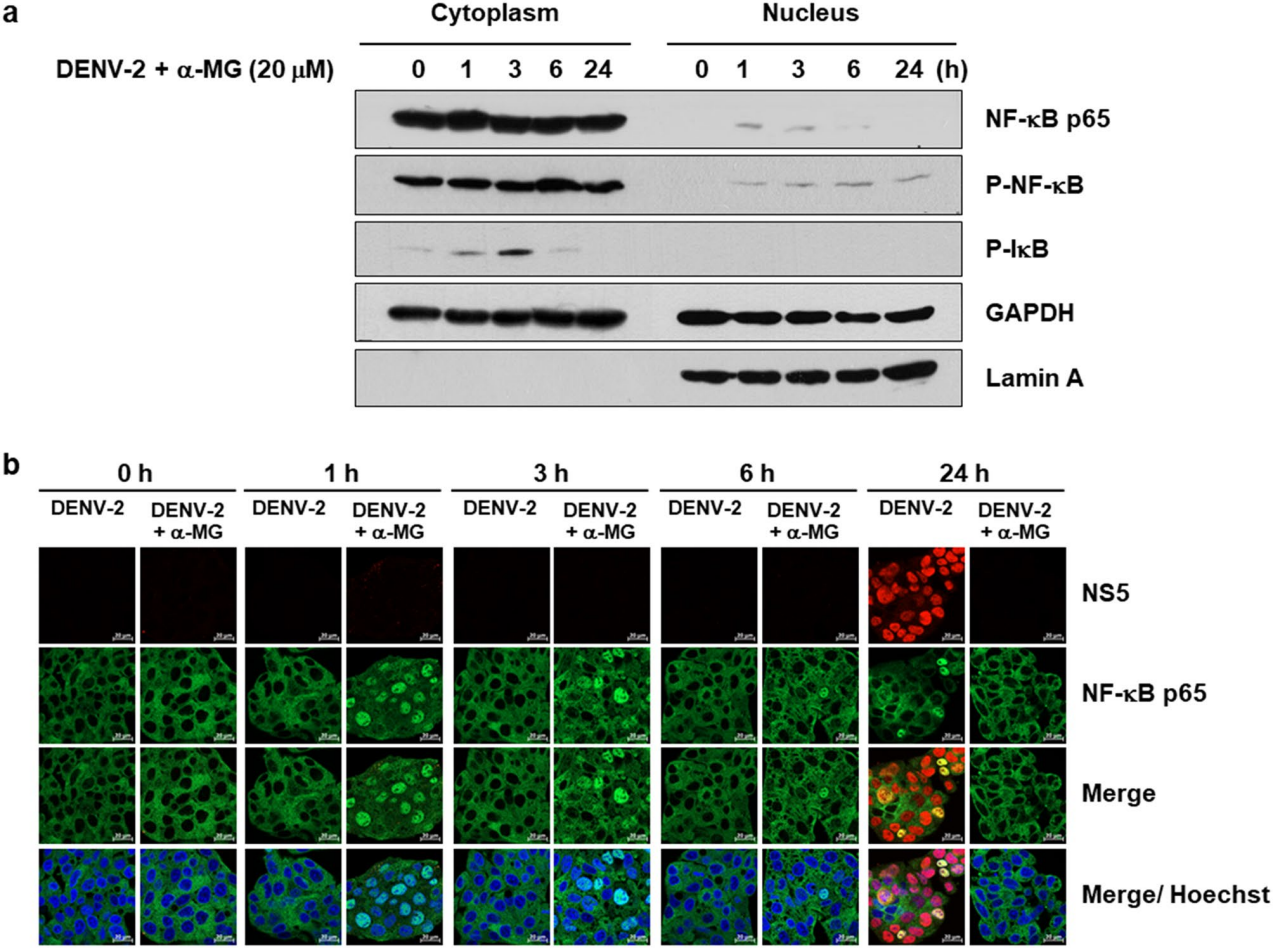
α-MG suppresses NF-κB activation. HepG2 were infected with DENV-2 at a MOI of 5 and treated with 20 μM of α-MG. The cells were harvested at 0, 1, 3, 6, and 24 h after treatment. (**a**) Cell lysates were separated into cytoplasmic and nuclear fractions. The expressions of NF-κB p65, phosphorylated NF-κB (P-NF-κB), and phosphorylated IκB (P-IκB) were determined by immunoblot assay using anti-NF-κB p65, anti-P-NF-κB, and anti-P-IκB antibodies, respectively. (**b**) Immunofluorescence assay demonstrates the expression and intracellular localization of NF-κB p65 (green) and DENV NS5 (red). Hoechst stain was used for nuclear staining (blue). Images are representative of three independent experiments.

To confirm that α-MG inhibited DENV-induced nuclear translocation of NF-κB p65, HepG2 cells were infected with DENV-2 and then incubated with 20 μM of α-MG. The cells were harvested at 0, 1, 3, 6, and 24 h post-treatment. The cytoplasmic and nuclear proteins were fractionated, and NF-κB p65, P-NF-κB, and P-IκB levels were examined by immunoblot assay. As shown in Fig. 7a and Fig. S4, α-MG treatment increased the levels of cytoplasmic and nuclear P-NF-κB at 1 h post-treatment, the increased levels were detected up to 6 h post-treatment, and a decreased level was still present at 24 h post-treatment. Treatment with α-MG also increased the levels of cytoplasmic P-IκB at 1 and 3 h post-treatment, a decreased level was still present at 6 h post-treatment, and no level was detected at 24 h post-treatment. The statistical analysis was determined to demonstrate the effect of α-MG on IκB degradation and phosphorylation of p65 at the indicated times post-treatment (Fig. S5). The results of immunofluorescence assay demonstrated increased levels of nuclear localization of NF-κB p65 at 1 and 3 h post-treatment of DENV-2-infected cells, but the levels of nuclear localization of NF-κB p65 were decreased at 6 and 24 h post-treatment (Fig. 7b). α-MG could induce NF-κB p65 activation in the presence of DENV-2 during the early phase of treatment and it was dramatically reduced by 24-h post-treatment. These results suggest that α-MG suppresses the NF-κB signaling pathway in DENV infection.

## Discussion

Viral and multiple host factors play a role in the progression of DENV infection to severe disease, such as dengue shock syndrome and dengue hemorrhagic fever. The main risk factor for severe disease is secondary infection, which was reported to be associated with high initial viral load and aberrant immune response^32–34^. Specific anti-DENV drugs are, therefore, needed to reduce viral load and cytokine storm, both of which are associated with disease severity and mortality. We hypothesized that an ideal anti-DENV drug would reduce both viral load and cytokine storm in DENV-infected patients. A previous study from our research group showed that a combination of the antiviral agent ribavirin (RV) and an anti-inflammatory agent (compound A [CpdA]) conferred greater efficiency for reducing DENV and cytokine/chemokine production in DENV-infected cells^35^. We later demonstrated that α-MG is a compound that contains both antiviral and anti-inflammatory properties against DENV infection^24^. α-MG exhibited broad-spectrum antiviral activity against all four DENV serotypes. In addition, α-MG manifested potent anti-inflammatory activity by reducing transcriptional responses of cytokines/chemokines observed in DENV-infected HepG2 cells^24^. Thus, α-MG has the potential to be further developed as an antiviral chemotherapy to control the spread of DENV, and to prevent the complications associated with DENV and the immunopathologic responses to DENV infection. However, the mechanisms of action of α-MG specific to the inhibition of DENV infection and cytokine/chemokine production have not yet been elucidated.

In this study, we investigated the molecular mechanisms underlying the antiviral and anti-inflammatory effects of α-MG in DENV infection. α-MG was shown to effectively reduce DENV infection and progeny virus production in many cell types. In addition to human hepatic HepG2 and Huh-7 cells^24^, antiviral activity of α-MG was confirmed in monocyte-derived dendritic cells (moDCs), peripheral blood mononuclear cells (PBMCs), and Vero CCL81 cells^36–38^. The IC50 at 24 h post-treatment in PBMCs was 5.47 μM^37^ and the IC50 in HepG2 and Huh-7 cells were 12.63 and 5.67 μM, respectively^24^. Among the cells that have been studied, hepatic cells, dendritic cells, and PBMCs have been most often studied because they are considered to be the major sites of DENV replication^39^. By way of example, liver injury is commonly observed in the severe forms of dengue infection^40^. In the present study, we repeated experiments from our previous study to show that α-MG at 20 μM significantly reduces DENV-infected cells and viral production in HepG2 cells. In addition, the viral NS5, NS1, and E proteins were downregulated after the DENV-infected HepG2 cells were treated with α-MG.

We performed a time-of-drug-addition assay in which HepG2 cells were treated with α-MG at preinfection, during infection, and postinfection with DENV-2 to identify the step at which α-MG acts during the DENV life cycle. The preinfection and during infection conditions reflect the actions of α-MG on virus cell binding and entry, whereas the postinfection condition reflects viral protein synthesis, RNA synthesis, virus assembly, and budding. The results of our experiments demonstrate the apparent effects of α-MG at the postinfection step. Infectious virus production was significantly inhibited when α-MG was added at 2, 4, and 6 hpi., while the inhibitory effect markedly decreased when α-MG was added at later time points (12 and 16 hpi), which suggests that α-MG might interfere with protein synthesis and/or RNA replication, but the early stage of the DENV life cycle was not affected. Time-of-drug-elimination assay was used to single out the step in which α-MG acts during the postinfection stage. α-MG treatment was significantly effective for reducing virus production and intracellular viral RNA when α-MG was added during 6–12 hpi, whereas the antiviral effect diminished when α-MG was added during 2–4 or 4–6 hpi. Thus, our data indicates that α-MG maximally inhibits the viral RNA synthesis step, but it does not affect the early stage (virus binding and entry), and the inhibitory effect markedly decreases at the late stage (virion assembly and budding) of the DENV life cycle. A similar observation was reported from a study that investigated the antiviral activity of α-MG in dendritic cells^36^.

NS5 is expected to be the potential target of α-MG during RNA replication because it carries enzymatic activities for viral RNA replication and is considered as a prime target for the design of antiviral inhibitors^8^. Moreover, previous study demonstrated α-MG to be responsible for suppression of HCV replication by inhibiting RNA polymerase activity of NS5B, which with it shares the characteristics and functions of DENV NS5^23^. Recent docking studies also showed that α-MG can interact with multiple DENV protein targets, such as NS5 RdRp, NS5 methyltransferase, NS2B-NS3 protease, and the glycoprotein E^38^. α-MG docked with strong binding affinity with NS5 RdRp and showed a potential binding site near the catalytic site interacting with the residues of all three conserved motifs (Q598-N614, G662-D664, and C709-R729), as well as with residues of the priming loop (H786-M809). However, direct interaction between α-MG and any DENV protein has never been validated. Our result in the present study demonstrated that α-MG effectively inhibits NS5 RdRp activity. We performed RdRp inhibition assay using a fluorescence-based transcription assay of purified DENV-2 RdRp protein. The formation of dsRNA from the single-stranded RNA poly(C) template was significantly reduced when recombinant RdRp protein was incubated with α-MG and detected by fluorescent dye PicoGreen. This result indicated that α-MG decreases DENV RNA synthesis by suppressing the activity of DENV NS5 RdRp (Fig. 3). The NS5 RdRp domain was demonstrated to interact with NS3^41^ and host nuclear transport proteins^42^. Thus, direct interaction of α-MG with NS5 RdRp possibly interrupts NS5 binding to a partner protein that can impact DENV replication in cells. NS3 helicase activity is required for unwinding of dsRNA and/or secondary structure of single-stranded RNA during viral RNA replication^43^. Apart from the interaction with viral NS3, the nuclear localization sequence (NLS) within the central region of DENV NS5 is recognized by host importin α/β for nuclear import of NS5 where it is thought to play a role in suppression of host antiviral response^42^. In particular, translocation of NS5 into the nucleus clearly associated with induction of RANTES expression by competitive binding to the host death-domain-associate protein (Daxx). Since NF-κB was bound to Daxx, the binding of NS5 and Daxx liberated NF-κB, which led to the increased likelihood of NF-κB to bind with the RANTES promoter resulting in increased RANTES expression^12,13^. Our results are consistent with those reported findings. In DENV-2-infected HepG2 cells, NS5 was predominantly present in the nucleus, there was a presence of nuclear NF-κB, and there was a high expression of RANTES. α-MG treatment in DENV-2-infected cells effectively reduced nuclear NS5 and nuclear NF-κB, and significantly suppressed the expression of RANTES. In addition to RANTES, IP-10, TNF-α, and IL-6 expression were also suppressed in α-MG treatment condition. RANTES, IP-10, TNF-α, and IL-6 were all reported to be regulated by NF-κB^44^. Reduction of nuclear NS5 after α-MG treatment could preserve the binding of Daxx and NF-κB, resulting in suppression of NF-κB-mediated cytokine/chemokine expression.

Studies of the infection cycle of DENV revealed that the cascade of inflammation is initiated in response to the DENV attack. DENV proteins, such as E protein, NS1, and NS5, are able to induce host inflammatory cytokines via NF-κB activation^5,6,11,12,45^. Our results show that α-MG treatment reduces viral RNA synthesis and subsequent viral protein production, including E protein, NS1, and NS5; therefore, this reduction of cytokine/chemokine production might be a consequence of reduced viral replication. A direct immunomodulatory effect of α-MG is also likely because the immunomodulatory and anti-inflammatory properties of α-MG were reported in various inflammatory disease models apart from virus infections, such as IL-1β-induced chondrocyte inflammation, LPS-induced brain inflammation, and LPS/D-GalN-induced acute liver failure^27–29^. Blocking of NF-κB activation and proinflammatory gene expression were mentioned as the underlying anti-inflammation mechanisms of α-MG. Ribavirin (RV) is a broad-spectrum antiviral agent that is active against a wide range of RNA viruses, and it has been used to treat chronic HCV infection. Our study used RV as an antiviral control and it demonstrated potent antiviral effect on DENV. Importantly, DENV-infected cells treated with α-MG had lower cytokine/chemokine levels than the DENV-infected cells treated with RV; however, α-MG treatment could not reduce infected cells, virus production, or viral proteins similar to the reductions effectuated by RV treatment (Figs. 1 and 5). In contrast and importantly, α-MG treatment yielded significant reduction in nuclear NF-κB p65 level that was comparable to the level of reduction caused by RV treatment (Fig. 6). We, therefore, hypothesized that there could be other mechanisms that give α-MG more anti-inflammatory activity than that exerted by RV in DENV infection. Since it is known that NF-κB regulates proinflammatory cytokine expression, we decided to investigate the NF-κB signaling pathway to evaluate inflammatory and immune responses in DENV infection in the presence or absence of α-MG. Our microarray results demonstrated that α-MG suppressed the expression of almost all of the antiviral and cytokine/chemokine genes that are regulated by NF-κB (Fig. 4). These results strongly suggest that α-MG inhibited DENV-induced inflammation via direct interaction with NF-κB or NF-κB-linked targets. Moreover, inhibition of DENV replication by α-MG is not related to upregulation of NF-κB-mediated antiviral IFN responses as is the case in many anti-DENV agents. For example, a natural product [(2 R,4 R)-1,2,4-trihydroxyheptadec-16-yne (THHY)] from avocado (*Persea americana*) fruit inhibits DENV replication via upregulation of NF-κB-dependent induction of antiviral interferon responses^46^. More specifically, NF-κB cascade is activated and IFN-α level is upregulated resulting in suppression of DENV replication. Alternatively, our results revealed that treatment with α-MG upon DENV infection could significantly downregulate NF-κB activation and reduce the expression levels of IFN-β1 (Figs. 4, 6 and 7). Furthermore, the anti-inflammatory effects of α-MG have been illustrated by various mechanisms. A recent study demonstrated that α-MG could inhibit cytokine secretion from T cells via the regulation of calcium signals on ORAI1 calcium channel and K+ channels^47^. However, the anti-inflammatory molecule that is directly affected by α-MG in DENV infection is not yet known and needs to be investigated.

**Figure 8:**
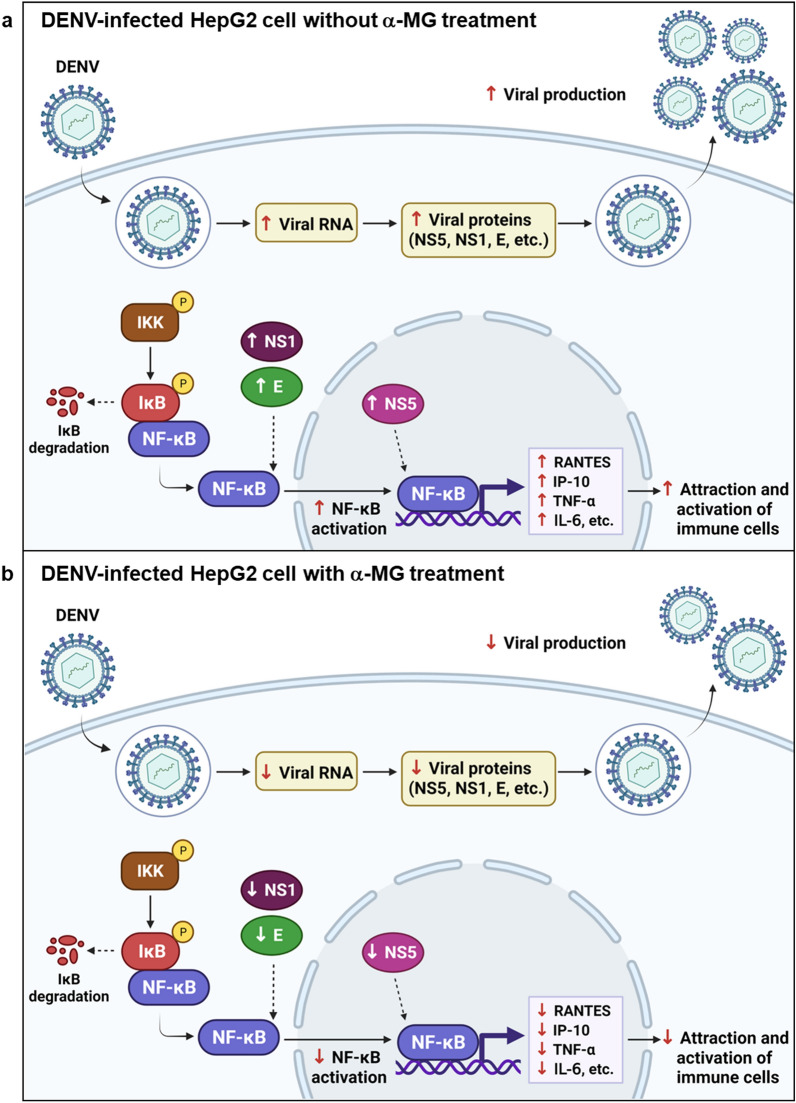
Proposed model for inhibition of dengue virus (DENV) and cytokine/chemokine production by α-MG.

In conclusion, α-MG is a promising anti-DENV agent due to its direct action against the virus and its inhibitory effect on the inflammatory mediators that are produced during DENV infection that are likely to be responsible for tissue damage and the systemic manifestations of DENV infection. Our findings demonstrated that α-MG could inhibit the replication step of the DENV life cycle. α-MG inhibited NS5 RdRp activity which play a vital role in viral RNA synthesis in DENV-infected cells leading to reduction of viral replication, viral protein production, and new infectious virion production. α-MG treatment reduced nuclear NS5 accumulation and NF-κB nuclear translocation resulting in the suppressed expression of the NF-κB-dependent cytokines/chemokines RANTES, IP-10, TNF-α, and IL-6. These findings indicate the ability of α-MG to directly inhibit DENV replication and modulate DENV-induced inflammatory responses (Fig. 8).

## Materials and methods

### Cell cultures and DENV propagation

Human hepatocellular carcinoma (HepG2, ATCC HB-8065) cells were grown in Dulbecco's Modified Eagle's Medium (DMEM) supplemented with 10% (v/v) fetal bovine serum (FBS), 2 mM L-glutamine, 1% (v/v) non-essential amino acids (NEAA), 1 mM sodium pyruvate, and 1% (v/v) penicillin–streptomycin in a 37 °C and 5% CO_2_ environment to determine the antiviral and anti-inflammatory activities of α-MG in DENV infection.

African green monkey kidney (Vero, ATCC CCL-81) cells were grown in Minimum Essential Medium (MEM) supplemented with 10% (v/v) FBS, 2 mM L-glutamine, and 1% (v/v) penicillin–streptomycin in a 37 °C and 5% CO_2_ environment for DENV titration by focus-forming unit (FFU) assay.

DENV-2 strain 16681 was propagated in *Aedes albopictus* C6/36 cell line (provided by the Armed Forces Research Institute of Medical Sciences, Thailand), which was cultured in Leibovitz’s L-15 Medium containing 1% FBS and 10% tryptose phosphate broth (TPB). Virus in the culture supernatant was titrated by FFU assay and maintained at − 80 °C until use.

Lenti-X™ 293T cells (Takara Bio, Inc., Shiga, Japan) and human cervical carcinoma (HeLa) cells (ATCC Cat# CRL-7923, RRID: CVCL_0030) were maintained in DMEM supplemented with 10% (v/v) FBS and 1% (v/v) of penicillin–streptomycin for DENV-2 RdRp expression and purification.

### DENV infection and compound treatment

To determine the antiviral activity of α-MG in DENV-infected HepG2 cells, cells were cultured in complete DMEM for 24 h and infected with DENV-2 at a multiplicity of infection (MOI) of 5. After adsorption at 37 °C for 2 h, unbound DENV were discarded and the HepG2 cells were washed three times with phosphate-buffered saline (PBS). They were then treated with medium containing either α-MG (Wako, Osaka, Japan), ribavirin (RV) (Sigma-Aldrich Corporation, St. Louis, MO, USA), dexamethasone (DEX) (Sigma-Aldrich), or ethanol (EtOH) solvent and incubated for 24 h. Non-DENV-infected cells were used as mock control. The treated cells were detected for DENV infection by flow cytometry. Culture supernatants were collected for determination of DENV production by FFU assay.

### Flow cytometry

To quantify the number of DENV-infected cells, mock and DENV-infected cells with or without treatment were detached with 0.1% trypsin and 2.5 mM EDTA in PBS, fixed with 4% paraformaldehyde in PBS for 20 min, and permeabilized with 0.2% Triton X-100 in PBS for 10 min. After washing, cells were stained with mouse monoclonal anti-DENV-E antibody (4G2) at 37 °C for 1 h followed by staining with Alexa Fluor 488-conjugated goat anti-mouse IgG (Molecular Probes, Eugene, OR, USA) for 30 min. Enumeration of DENV-infected cells was performed using a BD Accuri C6 Flow Cytometer (BD Biosciences, Franklin Lakes, NJ, USA) with 10,000-gated events per sample.

### Focus forming unit (FFU) assay

To examine the anti-DENV activity of α-MG on virus production, the newly synthesized virus in the culture supernatant was measured by FFU assay. Vero cells were plated in a 96-well plate (2 × 10^4^ cells per well) for 24 h. A ten-fold serial dilution of culture supernatant of mock and DENV infected cells, with or without treatment, was added to the cells. After 2 h-incubation at 37 °C, the infected cells were overlaid with 1.5% tragacanth gum (Sigma-Aldrich) in MEM with 2% FBS. The plate was then further incubated for 72 h. Cells were fixed with 3.8% formaldehyde in PBS for 10 min, and permeabilized with 1% Triton X-100 in PBS for 10 min. Cells were stained with mouse monoclonal anti-DENV-E antibody (4G2) at 37 °C for 1 h, and the wells were washed three times with PBS. The cells were then incubated with horseradish peroxidase (HRP)-conjugated rabbit anti-mouse IgG antibody (DAKO, Santa Clara, CA, USA) at a dilution of 1:1,000 in the dark for 1 h at room temperature (RT). DENV-infected cell foci were visualized by adding 0.6 mg/ml 3,3’-diaminobenzidine (DAB) substrate (Sigma-Aldrich) for 5–10 min at RT. The DENV foci were counted manually under an inverted light microscope (CKX53; Olympus Corporation, Tokyo, Japan).

### Quantitative real-time reverse transcription polymerase chain reaction (qRT-PCR) analysis

To quantitate the DENV RNA level, total RNA was extracted from mock and DENV-infected cells, with or without treatment, using TRIzol Reagent (Invitrogen Corporation, Carlsbad, CA, USA). In total, 450–1000 ng of purified RNA was used for reverse transcription to generate cDNA using AMV Reverse Transcriptase (Promega Corporation, Madison, WI, USA) according to the manufacturer’s instructions with minor modifications using a DENV NS1 reverse primer. Real-time PCR was set up using SYBR Green master mixes (Bio-Rad Laboratories, Hercules, CA, USA) in the presence of cDNA templates and specific primers for DENV NS1 (Table 1). DENV RNA standard at a known copy number/μl was also included in the reverse transcription and real-time PCR to serve as a standard curve for quantitation of the DENV RNA level^48^. Real-time PCR conditions that were set in the LightCycler 480 Instrument (Roche Diagnostics, Basel, Switzerland) included: (i) pre-incubation at 95 °C for 5 min; (ii) 45 amplification cycles of denaturation at 95 °C for 10 s, annealing at 55 °C for 10 s, and extension at 72 °C for 20 s; and, (iii) melting curve and cooling steps.

**Table 1:** Sequences of primers for the qRT-PCR analysis.

Primer	Orientation	Sequence (5’-3’)
NS1_F	Forward	GGAGACATCAAAGGAATCATGC
NS1_R	Reverse	GCCATCAATGAGAAAGGTCTGG
RANTES_F	Forward	TCCTGCAGAGGATCAAGACA
RANTES_R	Reverse	GTTATTTTCATTAGTGCCGCCGTGCCTTCTTTCGG
IP-10_F	Forward	GAATCGAAGGCCATCAAGAA
IP-10_R	Reverse	AAGCAGGGTCAGAACATCCA
TNF-α_F	Forward	TGCTTGTTCCTCAGCCTCTT
TNF-α_R	Reverse	ATGGGCTACAGGCTTGTCACT
IL-6_F	Forward	GTACATCCTCGACGGCATC
IL-6_R	Reverse	AGCCACTGGTTCTGTGCCT
β-Actin_F	Forward	AGAAAATCTGGCACCACACC
β-Actin_R	Reverse	CTCCTTAATGTCACGCACGA

To determine cytokine/chemokine transcription, total RNA was extracted from mock and DENV-infected cells, with or without treatment, using TRIzol reagent (Invitrogen). Cytokine/chemokine mRNA was quantified by real-time RT-PCR technique using specific primers (Table 1). Total RNA was reverse-transcribed into cDNA using Superscript III Reverse Transcriptase (Invitrogen). Amplification of cDNA by real-time PCR was performed in a reaction mixture of SYBR Green master mixes. The relative mRNA expression was normalized against the β-actin mRNA level using the comparative Ct method.

### Multiplexed bead-based immunoassays

To determine the effect of α-MG treatment on cytokine/chemokine secretion from DENV-infected HepG2 cells, cells were treated with α-MG or RV or DEX as previously described. The culture supernatants were collected at 24 h post-treatment and cytokine/chemokine levels of RANTES, IP-10, TNF-α, and IL-6 were analyzed by BD Accuri C6 Flow Cytometer using the BD CBA Human Soluble Protein Flex Set system (BD Biosciences), following the manufacturer’s instructions. Flow cytometry standard data files were analyzed by FCAP Array™ software (BD Biosciences) to generate standard curves, and to determine the concentration of unknown samples.

### Immunoblot analysis

To determine the effect of α-MG treatment on DENV-2 protein production, the DENV-infected cells were lysed with RIPA buffer (20 mM Tris–HCl pH7.5, 5 mM EDTA, 150 mM NaCl, 1% Triton X-100, 0.1% SDS, 0.5% deoxycholate, and protease inhibitor cocktail) for 30 min at 4 °C. Equal amounts of protein were separated using 10% SDS–polyacrylamide gel and transferred to nitrocellulose membranes. DENV NS5, NS1, and E proteins were detected using mouse anti-DENV-NS5 antibody (Invitrogen), mouse monoclonal anti-NS1 antibody (DN3) (Abcam, Cambridge, UK), and mouse monoclonal anti-DENV-E antibody (4G2) followed by HRP-conjugated rabbit anti-mouse IgG antibody (DAKO) at a dilution of 1:1,000, and then visualized with an enhanced chemiluminescence system (PerkinElmer, Waltham, MA, USA).

To determine the effect of α-MG treatment on NF-κB activation, cytoplasmic and nuclear proteins were fractionated using a Subcellular Protein Fractionation Kit (Thermo Fisher Scientific, Waltham, MA, USA). The expression of NF-κB p65 was determined by immunoblot technique with rabbit anti-NF-κB p65 antibody (Cell Signaling Technology, Danvers, MA, USA). The GAPDH protein was detected with anti-GAPDH antibody (Santa Cruz Biotechnology, Dallas, TX, USA), and its level served as an internal control. Lamin A was detected with mouse monoclonal anti-lamin A antibody (Abcam), and was used as a protein marker of nuclear fraction. NF-κB p65 bands were scanned and quantified using ImageJ software^49^.

To determine the effect of α-MG treatment on upstream signal transduction of NF-κB activation, HepG2 cells were infected with DENV-2 and treated with α-MG. The cells were harvested at 0, 1, 3, 6, and 24 h post-treatment. Cytoplasmic and nuclear proteins were fractionated. The expressions of NF-κB p65, phosphorylated NF-κB (P-NF-κB), and phosphorylated IκB (P-IκB) were detected with rabbit anti-NF-κB p65, rabbit anti-P-NF-κB, and rabbit anti-P-IκB antibodies (Cell Signaling Technology), respectively. The protein bands were scanned and quantified using ImageJ software^49^.

### Time-of-drug-addition and time-of-drug-elimination assays

To conduct the time-of-drug-addition assay, α-MG was added at different stages of DENV infection. HepG2 cells were seeded in 12-well plates (3.5 × 10^5^ cells/well). For pretreatment experiments, 20 μM of α-MG was added 2 h prior to infection. At the time of infection, the cells were infected with DENV-2 at an MOI 5 and cotreated with α-MG for 2 h. During the postinfection period, α-MG was added at 2, 4, 6, 12, and 16 hpi. In all experiments, the viruses were removed at 2 hpi, cells were washed twice with PBS, and then incubation was continued. At 24 hpi, cell supernatants were collected for FFU assay, and the cells were harvested for viral RNA copy quantification by qRT-PCR method.

Time-of-drug-elimination assay was performed to identify the action of α-MG during the postinfection steps of the DENV life cycle. HepG2 cells were infected with DENV-2 at an MOI 5. At 2 hpi, the cells were washed twice with PBS, and then 20 μM of α-MG was added during 2–4, 4–6, and 6–12 hpi. At 24 hpi, cell supernatants were collected for FFU assay, and the cells were harvested for DENV RNA quantification by qRT-PCR method.

### Expression and purification of DENV-2 RdRp protein

Recombinant DENV-2 RdRp was expressed and purified as described previously with modification^31^. The DENV-2 RdRp-encoding region (a.a. 251–896) (GenBank: U87411.1) was incorporated into the lentiviral transfer pcDH vector, with N-terminal signal peptide of albumin protein sequence and C-terminal tobacco etch virus (TEV) protease cleavage site, human influenza hemagglutinin epitope (HA) tag, and 6xHistidine tag sequences. The sequences of the primers used in this construction are shown in Supplementary Table 3. The correctness of the sequence of DENV-2 RdRp construct was verified by Sanger sequencing. Lentiviral vector carrying DENV-2 RdRp gene was produced by transfection of the transfer pcDH-DENV-2 RdRp plasmid along with lentiviral accessory gene psPAX2 plasmid and VSV-G pseudotype pMD.G2 plasmid into Lenti-X™ HEK293T cell line and then transduced into HeLa cells^50^. The stable DENV-2 RdRp expressing cells were selected with puromycin. The expression of DENV-2 RdRp was further verified by indirect immunofluorescence assay and immunoblot analysis using an anti-HA antibody (Invitrogen). Polyhistidine-tagged-DENV-2 RdRp was purified by TALON™ Metal Affinity Resin (Takara Bio) under native conditions. The purified protein was concentrated and buffer exchanged by ultrafiltration tubes into RdRp reaction buffer. Protein concentration was determined by using Bradford dye reagent (Bio-Rad Laboratories).

### DENV-2 RdRp inhibition assay

RdRp assay was adapted from a published protocol with modifications^31^. In vitro RNA synthesis assays were performed within 96-well black plates using a single-stranded RNA poly(C) as template and GTP as substrate in a 25 μl reaction mixture. Briefly, 4 μl of DENV-2 RdRp (12.5 μM) was mixed with either 1 μl of various concentrations of α-MG or the compound vehicle EtOH in RdRp reaction buffer containing 20 mM Tris–HCl (pH 7.4), 11.9 mM NaCl, 7.5% glycerol, 5 mM MnCl_2_, and 7 mM 2-mercaptoethanol. Subsequently, 1 μl of 1 mg/ml poly(C) (Sigma-Aldrich) and 2 μl of 4 mM GTP were added into the mixture. The final concentrations of the reaction components were 2 μM DENV-2 RdRp, 40 μg/ml poly(C), 320 μM GTP, 0–40 μM α-MG, 20 mM Tris–HCl (pH 7.4), 11.9 mM NaCl, 7.5% glycerol, 5 mM MnCl_2_, and 7 mM 2-mercaptoethanol. After incubating at 37 °C for 60 min, the formation of double-stranded RNA (dsRNA) was quantitated by adding 85 μl of fluorescent dye PicoGreen (Thermo Fisher Scientific), diluted 350-fold with TE buffer, into each well. Fluorescence was measured with Synergy H1 multi-mode microplate reader (Biotek Instruments, Winooski, VT, USA) at excitation and emission wavelengths of 485 nm and 510 nm, respectively. The RdRp activity for each well was calculated as follows: Inhibition (%) = 100 × (experimental read value–positive control average value)/(negative control average value–positive control average value), where the positive control value was obtained with 0.82% EtOH (without α-MG) in the presence of poly(C), while negative control value was obtained with 0.82% EtOH (without α-MG) in the absence of poly(C). The IC50 value was determined by nonlinear regression, variable slope model in GraphPad Prism.

### Real-time PCR array

Human Antiviral Response RT^2^ Profiler PCR Array and Human Cytokines and Chemokines RT^2^ Profiler PCR Array (QIAGEN, Hilden Germany) were selected for analysis of gene expression in response to DENV infection and cytokine/chemokine production, respectively. Total RNA was isolated either from mock-infected HepG2 cells as control cells or from DENV-2-infected cells in the presence or absence of 20 μM of α-MG using an RT^2^ First Strand Kit (QIAGEN). For RNA quality control, the RNA concentration and ribosomal RNA band integrity of each sample were measured by Qubit 2.0 (Invitrogen) and an Agilent Bioanalyzer 2100 (Agilent Technologies), respectively. One microgram (μg) of RNA sample was transcribed into cDNA using an RT^2^ First Strand Kit (QIAGEN). The cDNA was further mixed with SYBR Green RT^2^ qPCR Mastermix (QIAGEN), and equal volumes were aliquoted into the wells of the PCR arrays containing 84 antiviral response-related genes, and 84 cytokine and chemokine genes. The PCR amplification steps were performed in a Roche LightCycler 480 instrument (Roche Diagnostics) and the Ct values were analyzed using the web-based program http://pcrdataanalysis.sabiosciences.com/pcr/arrayanalysis.php for 2^−ΔΔCt^ analysis. The results are presented as fold increase or decrease relative to the results of mock-infected HepG2 cells.

### Indirect immunofluorescence assay

To determine the influence of α-MG on the presence of DENV-2 NS5 and nuclear translocation of NF-κB p65, HepG2 cells were grown on glass coverslips for 24 h and infected with DENV-2 in the presence or absence of α-MG or RV as described above. After incubation at 37 °C in a 5% CO_2_ atmosphere for 24 h, the treated cells were fixed with 4% paraformaldehyde in PBS, permeabilized with 0.2% Triton X-100 in PBS, and incubated with a mixture of mouse anti-DENV-NS5 antibody and rabbit anti-NF-κB p65 at RT for 1 h. After washing with PBS, the cells were incubated with a mixture of Cy3-conjugated goat anti-mouse IgG (Invitrogen), Alexa Fluor 488-conjugated donkey anti-rabbit IgG (Invitrogen), and Hoechst 33342 for nuclear staining (Invitrogen) at RT for 1 h. Fluorescence images were visualized using a laser scanning confocal microscope (LSM 800; Zeiss Microscopy, Jena, Germany).

To investigate the influence of α-MG on the presence of DENV-2 NS5 and nuclear translocation of NF-κB p65 during the early phase of infection, HepG2 cells were infected with DENV-2 and treated with 20 μM of α-MG. The infected cells were harvested at 0, 1, 3, 6, and 24 h post-treatment. DENV-2 NS5 and NF-κB p65 were detected as described above.

### Statistical analysis

All statistical analyses were performed using Graph-Pad Prism 6 Software (GraphPad Software, Inc., San Diego, CA, USA). All experiments were performed in triplicate, and the results are presented as mean ± standard deviation (SD). Statistical differences between groups were analyzed by one-way analysis of variance (ANOVA), followed by Tukey's honestly significant difference (HSD) test. Two-way ANOVA and Bonferroni posttest were used to determine differences between groups that were split on two factors. A p-value less than 0.05 was considered to be statistically significant for all tests.

## Supplementary Information

Supplementary Information.

## Data Availability

All data generated or analyzed during this study are included in this published article and its supplementary information file.
