# Supplementary material for: Alpha-mangostin inhibits viral replication and suppresses nuclear factor kappa B (NF-κB)-mediated inflammation in dengue virus infection
Source: Sci Rep. 2022 Sep 27;12:16088. doi: 10.1038/s41598-022-20284-7 (PMC9515165; doi:10.1038/s41598-022-20284-7)
Supplement: Supplementary file 1 — Supplementary Information. [file 41598_2022_20284_MOESM1_ESM.pdf]

Alpha-mangostin inhibits viral replication and suppresses nuclear factor kappa B (NF-κB)-mediated inflammation in dengue virus infection

Mayuri Tarasuk[1], Pucharee Songprakhon[2], Thaweesak Chieochansin[2], Kornkan Choomee[2], Kesara Na-Bangchang[1,3], Pa-thai Yenchitsomanus[2*]

[1]Graduate Program in Bioclinical Sciences, Chulabhorn International College of Medicine, Thammasat University, Pathum Thani, Thailand

[2]Division of Molecular Medicine, Research Department, Faculty of Medicine Siriraj Hospital, Mahidol University, Bangkok, Thailand

[3]Drug Discovery and Development Center, Thammasat University, Pathum Thani, Thailand

Correspondence to:

Pa-thai Yenchitsomanus, PhD

Professor of Medical Genetics and Immunology

Division of Molecular Medicine, Research Department

Faculty of Medicine Siriraj Hospital, Mahidol University

2 Wanglang Road, Bangkoknoi, Bangkok 10700, Thailand

Tel: (+66) 2-419-2777; Fax: (+66) 2-411-0169

E-mail: pathai.yen@mahidol.ac.th, ptyench@gmail.com

Supplementary Information

Supplementary Table 1. Fold changes of antiviral gene expression in DENV-infected HepG2 cells in the presence or absence of 20 μM of α-MG

Gene abbreviation	Formal gene name	Fold changes	
		**DENV-2 + EtOH**	**DENV-2 + α-MG**
**CCL5**	Chemokine (C–C motif) ligand 5	1,509.65	265.03
**IFNB1**	Interferon beta, beta 1, fibroblast	362.04	34.06
**CXCL10**	Chemokine (C–X–C motif) ligand 10	349.71	25.99
**TNF**	Tumor necrosis factor	171.25	3.25
**OAS2**	2'-5'-Oligoadenylate synthetase 2	114.56	15.78
**CXCL11**	Chemokine (C–X–C motif) ligand 11	30.48	4.53
**CCL3**	Chemokine (C–C motif) ligand 3	29.04	1.31
**CXCL8**	Chemokine (C–X–C motif) ligand 8	27.28	6.87
**MX1**	Myxovirus (influenza virus) resistance 1, interferon-inducible protein p78 (mouse)	12.38	2.85
**FOS**	FBJ murine osteosarcoma viral oncogene homolog	7.36	2.75
**ISG15**	ISG15 ubiquitin-like modifier	6.54	2.10
**IFIH1**	Interferon induced with helicase C domain 1	6.15	1.85
**NFKBIA**	Nuclear factor of kappa light polypeptide gene enhancer in B-cells inhibitor, alpha	5.10	1.14
**DHX58**	DEXH (Asp-Glu-X-His) box polypeptide 58	3.66	2.25
**DDX58**	DEAD (Asp-Glu-Ala-Asp) box polypeptide 58	2.97	1.95
**IL6**	Interleukin 6	2.89	1.17
**NOD2**	Nucleotide-binding oligomerization domain-containing protein 2	2.85	0.69
**TLR-3**	Toll-like receptor 3	2.81	0.41
**IL15**	Interleukin 15	2.75	0.67
**STAT1**	Signal transducer and activator of transcription 1	2.38	1.37
**CYLD**	Cylindromatosis (turban tumor syndrome)	2.35	1.11
**JUN**	Jun proto-oncogene	2.25	1.21
**IL18**	Interleukin 18	2.20	2.79
**MEFV**	Mediterranean fever	2.20	0.72
**CXCL9**	Chemokine (C–X–C motif) ligand 9	2.13	1.61

Values represent mRNA fold changes of antiviral genes in DENV-infected HepG2 cells in the presence or absence of 20 μM of α-MG relative to mock-infected cells

Abbreviations: DENV, dengue virus; HepG2 cells, human hepatocellular carcinoma cells; α-MG, alpha-mangostin

Supplementary Table 2. Fold changes of cytokine and chemokine gene expression in DENV-infected HepG2 cells in the presence or absence of 20 μM of α-MG

Gene abbreviation	Formal gene name	Fold changes	
		DENV-2 + EtOH	DENV-2 + α-MG
**CCL5**	Chemokine (C–C motif) ligand 5	1,039.73	191.34
**CXCL10**	Chemokine (C–X–C motif) ligand 10	270.97	13.09
**TNF**	Tumor necrosis factor	117.95	6.87
**CXCL2**	Chemokine (C–X–C motif) ligand 2	45.00	3.27
**LTB**	Lymphotoxin beta (TNF superfamily, member 3)	28.48	1.75
**CXCL8**	C-X-C motif chemokine ligand 8 (Interleukin 8)	26.95	5.70
**CXCL11**	Chemokine (C–X–C motif) ligand 11	20.14	2.33
**CCL3**	Chemokine (C–C motif) ligand 3	15.05	1.35
**CXCL1**	Chemokine (C–X–C motif) ligand 1	13.76	2.11
**CSF3**	Colony stimulating factor 3	10.00	2.00
**IL23A**	Interleukin 23, alpha subunit p19	6.11	1.97
**CCL20**	Chemokine (C–C motif) ligand 20	5.66	1.46
**IL11**	Interleukin 11	4.67	3.20
**OSM**	Oncostatin M	3.08	1.38
**BMP6**	Bone morphogenetic protein 6	3.04	5.17
**LIF**	Leukemia inhibitory factor	2.64	1.68
**TGFB2**	Transforming growth factor, beta 2	2.59	0.88
**IL6**	Interleukin 6	2.54	0.85
**CXCL5**	Chemokine (C–X–C motif) ligand 5	2.32	0.75
**IL15**	Interleukin 15	2.30	0.74
**LTA**	Lymphotoxin alpha (TNF superfamily, member 1)	2.02	1.17

Values represent mRNA fold changes of cytokine and chemokine genes in DENV-infected HepG2 cells in the presence or absence of 20 μM of α-MG relative to mock-infected cells

Abbreviations: DENV, dengue virus; HepG2 cells, human hepatocellular carcinoma cells; α-MG, alpha-mangostin

Supplementary Table 3. Primers used for DENV-2-RdRp construction

Primer	Orientation	Sequence (5'-3')
DENV-2 RdRp_F	Forward	GGGTCGCGGATCCACTTACGAGCCAGATGTAGACCTCGG
DENV-2 RdRp_R	Reverse	TCGAGTGCGGCCGCGCCCTGAAAATACAGGTTTTCTGCCTCTTCCTCTTCTCTTCTGAATCTTTTCATG

Original immunoblot for Fig.1

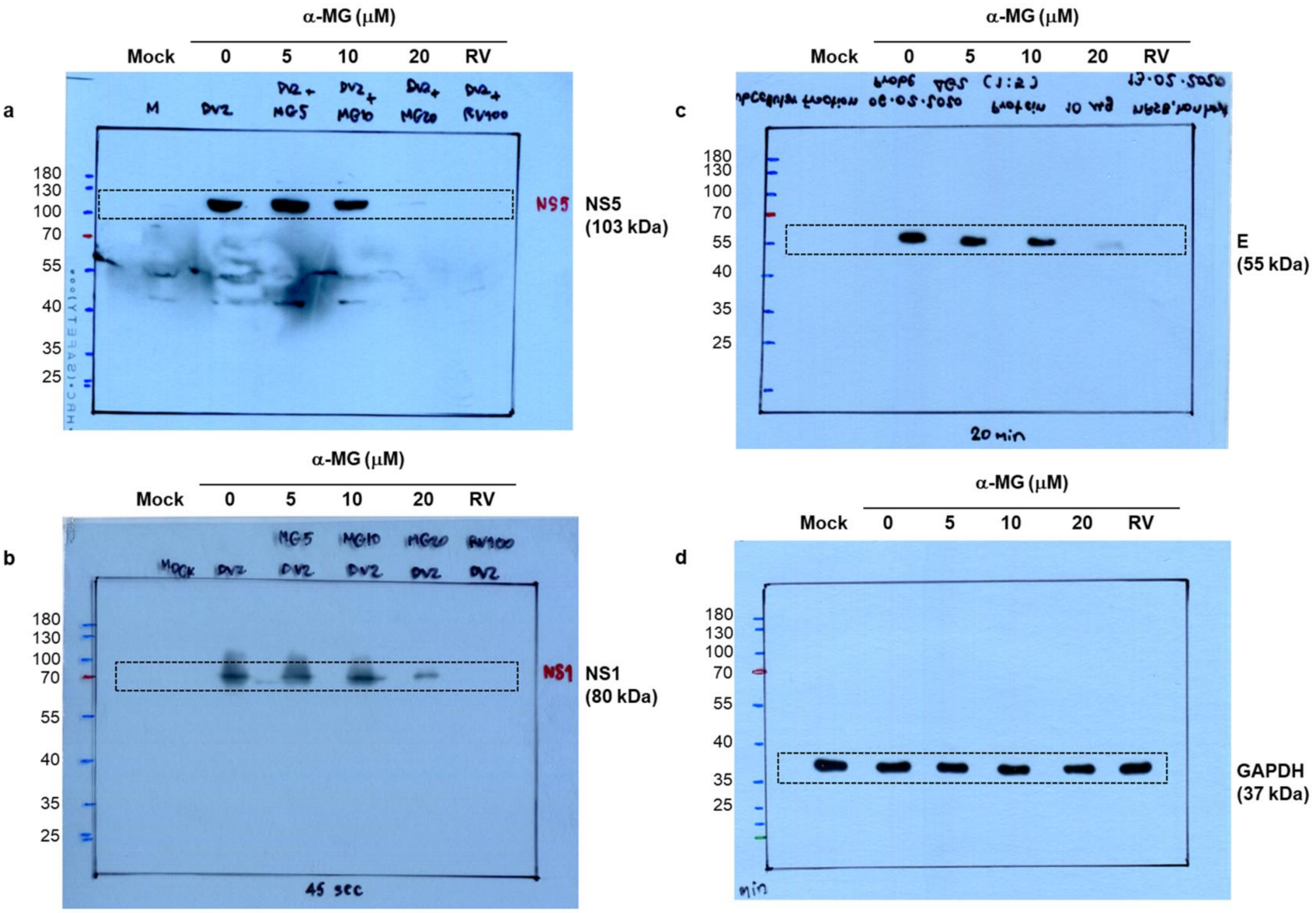

Fig. S1 Immunoblot images with cropped areas indicated by dashed lines. The results are the representatives from three independent experiments. The samples were obtained from the same experiment and the blots were processed in parallel. Each membrane was separately incubated with specific antibody to detect either (a-c) DENV NS5, NS1, or E proteins. (d) The same membrane was incubated with anti-GAPDH antibody.

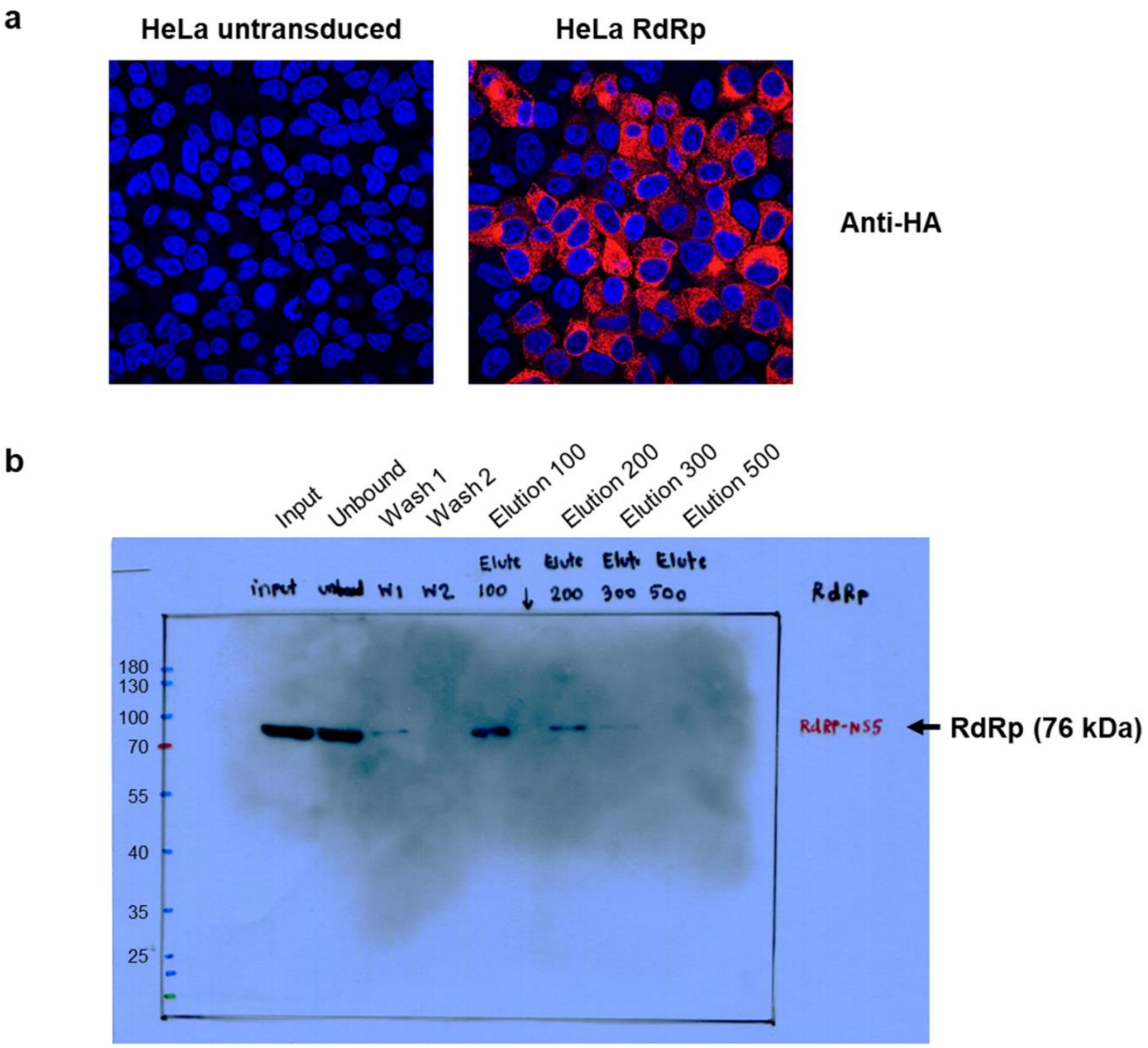

Fig. S2 Expression and purification of DENV-2 RdRp. (a) HeLa cells expressing stable DENV-2 RdRp are shown by indirect immunofluorescence assay using anti-HA antibody and Cy3-conjugated goat anti-mouse IgG antibody (red). The HeLa untransduced cells were used as control. (b) Immunoblot analysis for determining the purified recombinant DENV-2 RdRp protein. The protein fractions were subjected to electrophoresis and the purified DENV-2 RdRp protein was detected using anti-HA antibody and HRP-conjugated rabbit anti-mouse IgG antibody. The histidine-tagged DENV-2 RdRp protein was eluted with 100, 200, 300, and 500 mM imidazole.

Original immunoblot for Fig.6

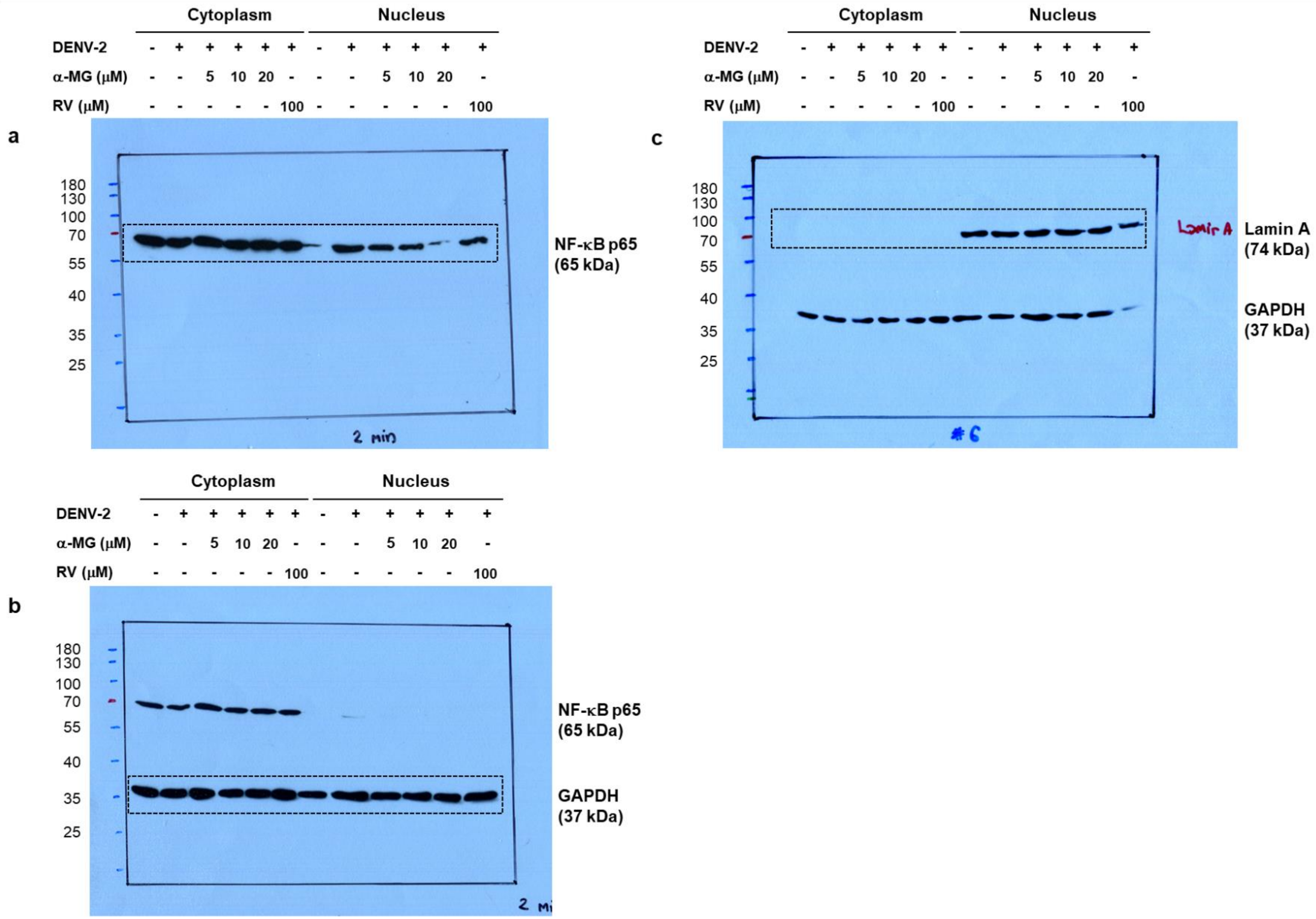

Fig. S3 Immunoblot images with cropped areas indicated by dashed lines. The results are the representatives from three independent experiments. Quantitative comparisons between samples were performed on the same blot. (a) The membrane was incubated with anti-NF-κB p65 antibody. (b) The same membrane was incubated with anti-GAPDH antibody. (c) The samples were obtained from the same experiment, the blot was processed in parallel, and the membrane was incubated with anti-lamin A antibody. Then, the membrane was incubated with anti-GAPDH antibody.

Original immunoblot for Fig.7

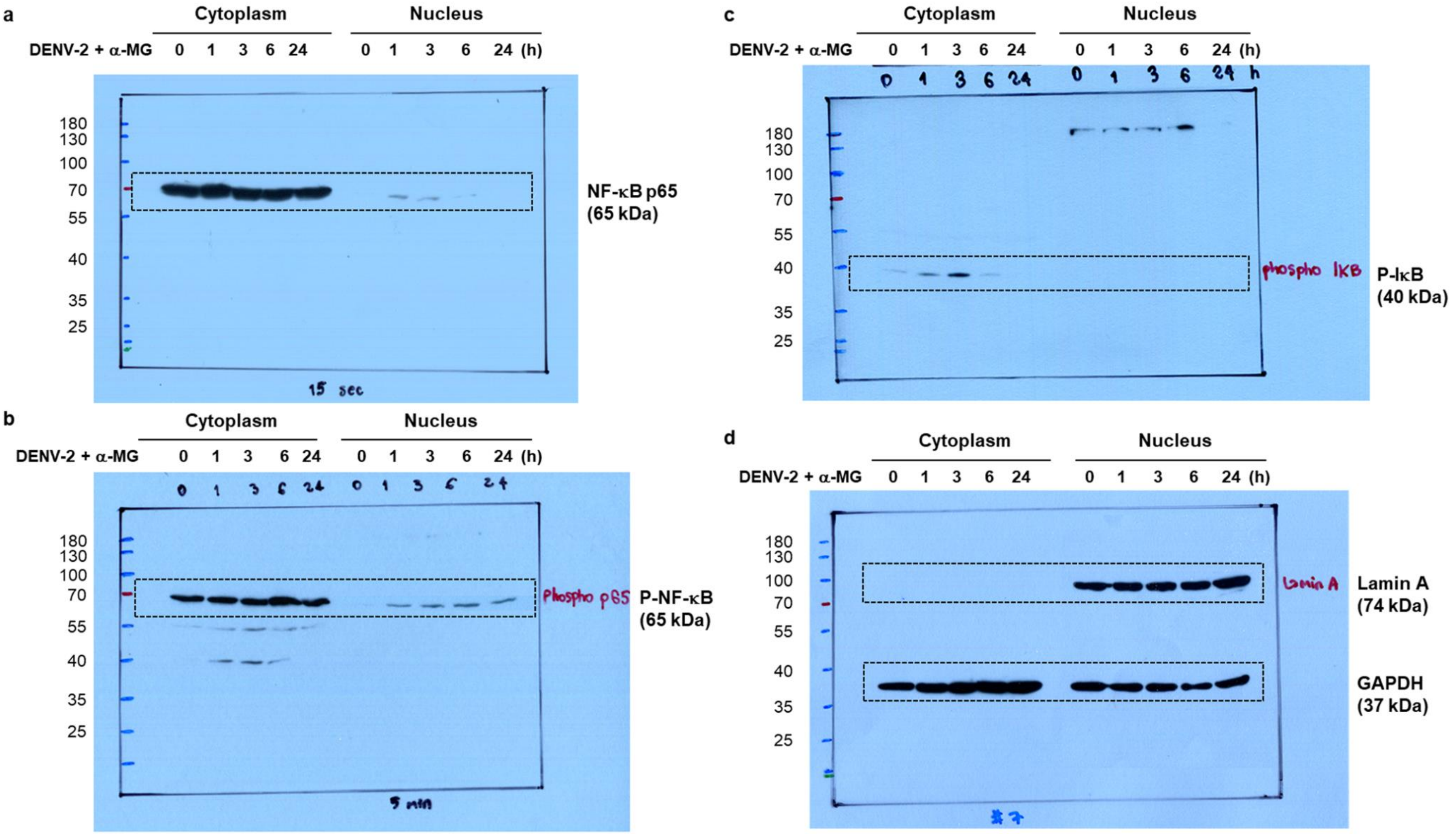

Fig. S4 Immunoblot images with cropped areas indicated by dashed lines. The results are the representatives from three independent experiments. The samples were obtained from the same experiment and the blots were processed in parallel. Each membrane was separately incubated with specific antibody to detect either (a) NF-κB p65, (b) P-NF-κB p65, or (c) P-IκB. (d) The membrane was incubated with anti-lamin A and anti-GAPDH antibodies to detect lamin A and GAPDH.

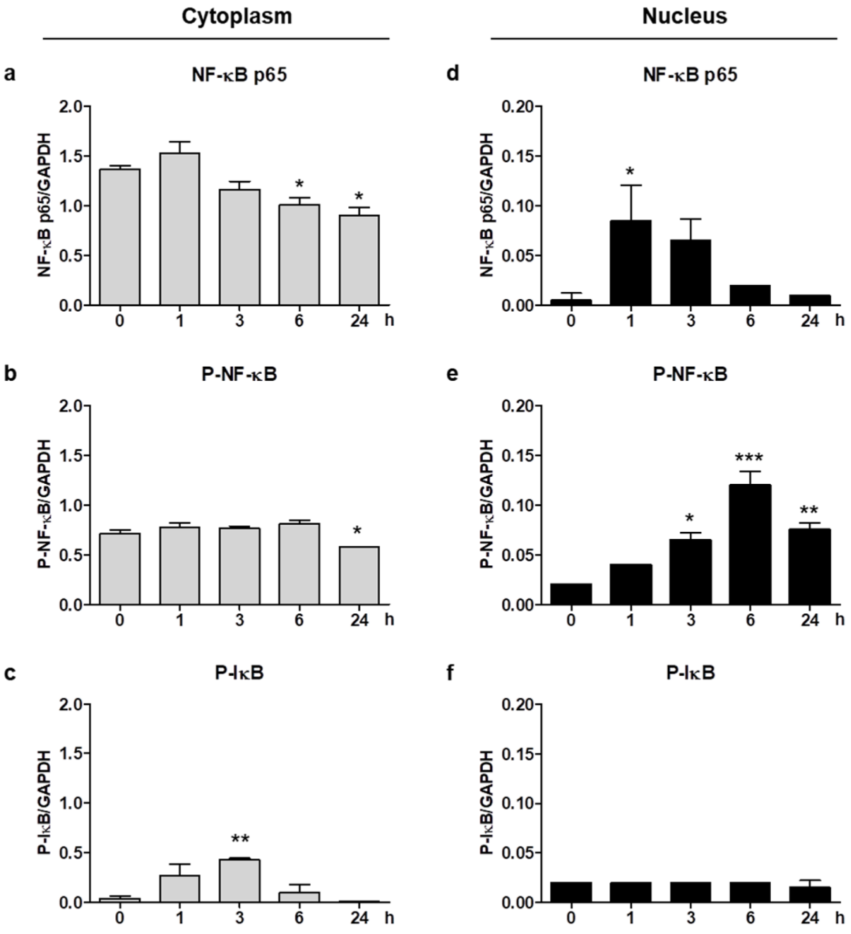

Fig. S5 α-MG suppresses NF-κB activation. HepG2 were infected with DENV-2 at a MOI of 5 and treated with 20 μM of α-MG. The cells were harvested at 0, 1, 3, 6, and 24 h after treatment. Cell lysates were separated into cytoplasmic and nuclear fractions. The expressions of NF-κB p65, phosphorylated NF-κB (P-NF-κB), and phosphorylated IκB (P-IκB) were determined by immunoblot assay using anti-NF-κB p65, anti-P-NF-κB, and anti-P-IκB antibodies, respectively (Fig. 7a). The optical densities normalized to GAPDH are shown. One-way ANOVA followed by Tukey's HSD test were used to evaluate for differences in protein levels as compared to 0 h post- treatment of α-MG (*p<0.05, **p<0.01, ***p<0.001).
